# A drug response prediction method for single-cell tumors combining attention networks and transfer learning

**DOI:** 10.3389/fmed.2025.1631898

**Published:** 2025-08-21

**Authors:** BingWei Zhou, SiLin Sun, ShengZheng Liu, HaiXia Long, YuChun Li

**Affiliations:** ^1^College of Information Science and Technology, Hainan Normal University, Guilinyang Campus, Haikou, Hainan, China; ^2^Key Laboratory of Data Science and Smart Education, Ministry of Education, Hainan Normal University, Haikou, China

**Keywords:** tumor drug response prediction, attention mechanism, transfer learning, bulk RNA sequencing, single-cell RNA sequencing

## Abstract

**Introduction:**

Accurately predicting tumor cell line responses to therapeutic drugs is essential for personalized cancer treatment. Current methods using bulk cell data fail to fully capture tumor heterogeneity and the complex mechanisms underlying treatment responses.

**Methods:**

This study introduces a novel method, ATSDP-NET (Attention-based Transfer Learning for Enhanced Single-cell Drug Response Prediction), which combines bulk and single-cell data. The model utilizes transfer learning and attention networks to predict drug responses in single-cell tumor data, after pre-training on bulk cell gene expression data. A multi-head attention mechanism is incorporated to enhance the model's expressive power and prediction accuracy by identifying gene expression patterns linked to drug reactions.

**Results:**

ATSDP-NET outperforms existing methods in drug response prediction, as demonstrated on four single-cell RNA sequencing datasets. The model showed superior performance across multiple metrics, including recall, ROC, and average precision (AP). It accurately predicted the sensitivity and resistance of mouse acute myeloid leukemia cells to I-BET-762 and the sensitivity and resistance of human oral squamous cell carcinoma cells to cisplatin. Correlation analysis revealed a high correlation between predicted sensitivity gene scores and actual values (R = 0.888, *p* < 0.001), while resistance gene scores also showed a significant correlation (R = 0.788, *p* < 0.001). The dynamic process of cells transitioning from sensitive to resistant states was visualized using uniform manifold approximation and projection (UMAP).

**Discussion:**

ATSDP-NET identifies critical genes linked to drug responses, confirming its predictions through differential gene expression scores and gene expression patterns. This method provides valuable insights into the mechanisms of drug resistance and offers potential for developing personalized treatment strategies.

## 1 Introduction

Understanding how individual cancer cells respond to drugs is critical for precision oncology. In this study, we aim to predict the drug response—specifically, whether a cell will survive or dieusing single-cell RNA sequencing (scRNA-seq) data collected before treatment. Our analysis is performed entirely at the single-cell level, rather than at the level of whole patients or animal models. While clinical decision-making depends on patient-level outcomes, modeling drug response at the cellular level serves as a necessary intermediate step toward precision medicine. It allows us to uncover cellular heterogeneity and understand how specific cell populations contribute to overall treatment response.

The rapid advancement of pharmacogenomics has led to the development of essential resources such as the cancer cell line encyclopedia (CCLE) (1) and the genomics of drug sensitivity in cancer (GDSC) (2, 3). Understanding the molecular mechanisms behind treatment sensitivity and resistance has advanced significantly thanks to these databases, which offer thorough genomic and drug response (4) data across a variety of cancer cell lines (5). Through the utilization of these databases, scientists are able to pinpoint important genomic factors that impact medication response, providing vital information to guide the creation of more efficient and customized cancer treatment plans.

Understanding and addressing the complexity of cancer genetics has evolved significantly with the introduction of precision medicine, which has further enhanced the development of tailored cancer treatment methods based on patient-specific data (6). Notably, the development of single-cell RNA sequencing (scRNA-seq) technology has opened up hitherto unheard-of possibilities for identifying cellular heterogeneity in malignancies (7) and clarifying drug response pathways. By enabling predictions at the single-cell level (8, 9), scRNA-seq facilitates the detailed examination of cancer cell responses to therapeutic agents. This method provides strong assistance for the development of precision medicine by taking into account structural differences in the data (10) and highlighting the heterogeneity present in single-cell data.

Because of their inherent complexity and heterogeneity, scRNA-seq data (11–15) provide special computational challenges despite their revolutionary promise. The inability of current techniques to accurately represent this complexity highlights the pressing need for innovative strategies that can more accurately anticipate and interpret drug responses at the single-cell level.

Drug response prediction has made substantial use of deep learning models, including convolutional neural networks (CNNs) and recurrent neural networks (RNNs) (16). Predicting the response to cancer drugs is made possible by these models' exceptional ability to spot intricate, non-linear patterns in vast amounts of biological data. Aliper et al. (17) successfully applied deep learning to predict cancer drug responses (17), and Xia et al. (18) employed deep neural networks to predict drug sensitivity across various cancer cell lines, demonstrating the promise of deep learning in this domain (18). Furthermore, Chiu et al. (19) developed a multimodal deep learning framework that combined gene expression data and drug structure to enhance predictive performance (19). Nevertheless, even with these developments, current deep learning models are unable to adequately handle important issues like data imbalance and small sample sizes, especially when it comes to a variety of cancer subtypes (20).

Transfer learning, which uses information from similar tasks or domains to improve model generalization in cancer treatment response prediction, has become a successful tactic to solve these issues. Through the use of sizable, labeled datasets, transfer learning enables better performance in situations with less data. Pan and Yang (21) introduced the concept of transfer learning, which has been widely applied in biomedical research. Subsequently, Tan et al. (22) optimized deep transfer learning frameworks to address issues such as feature misalignment (22), and Zhu et al. (23) proposed an integrated transfer learning framework that enhanced the performance of drug response prediction models, particularly for novel tumor cells and drugs. Nevertheless, the limited availability of single-cell data frequently limits the efficiency of transfer learning techniques (24) in real-world applications, making it difficult for them to produce predictions for single-cell RNA-seq data that are particular to a certain cell type.

Recent studies in the area of single-cell drug response prediction have explored novel approaches using deep learning and transfer learning. One such approach is scDEAL (25), which uses bulk-to-single-cell transfer learning to enhance drug response predictions at the single-cell level. Although similar in its use of transfer learning, our proposed ATSDP-NET model introduces a multi-head attention mechanism, which improves the precision and interpretability of drug response predictions. Similarly, CSG2A (26) predicts drug response heterogeneity using single-cell transcriptomic signatures. While this approach focuses on therapeutic vulnerabilities, our method specializes in predicting drug response at the single-cell level, offering a more detailed and targeted analysis. In contrast, the THERAPI framework (27) provides a model for predicting therapeutic responses in cancer treatment. However, unlike our approach, THERAPI does not incorporate the multi-head attention mechanism, which is a key component in ATSDP-NET, allowing for a finer resolution of drug response heterogeneity at the single-cell level. Furthermore, the study by Hsu et al. (28) uses a deconvolution approach for predicting drug response by analyzing tumor and cancer cell lines. While this method provides valuable insights into drug response, it lacks the integration of multi-head attention, which limits its ability to capture the complex heterogeneity of single-cell RNA-seq data.

These works provide important context for our research, highlighting the evolution of drug response prediction methods. To get around these restrictions, we introduce a multi-head attention mechanism within the transfer learning framework. This will help the model better capture important characteristics of single-cell RNA-seq data and increase the accuracy of its predictions. The attention mechanism increases the model's sensitivity to particular characteristics of single-cell data by allowing it to pinpoint important elements pertinent to drug response at the single-cell level. To extract important features utilizing multi-head attention and transfer learning, we provide ATSDP-NET (Attention-based Transfer Learning for Enhanced Single-cell Drug Response Prediction), a unique system that combines bulk and single-cell RNA-seq data. Superior prediction accuracy and interpretability are attained by ATSDP-NET by the identification of important genes linked to drug response.

Extensive evaluations of scRNA-seq datasets show that ATSDP-NET performs well in predicting drug responses in a range of cell types. Additionally, tests on several benchmark scRNA-seq datasets treated with various medications demonstrate its remarkable predictive power. Through the identification of important gene profiles associated with drug response, ATSDP-NET advances our knowledge of drug response at the single-cell level and offers fresh perspectives on the molecular mechanisms underlying drug sensitivity and resistance.

Our proposed method aims not only to improve single-cell level drug response prediction but also to provide a foundational step toward personalized cancer treatment by modeling tumor heterogeneity more effectively. While the use of single-cell data provides detailed insights into drug response at the cellular level, we acknowledge that directly linking these findings to patient-level precision medicine requires further steps. In future work, we plan to integrate patient-derived data or pseudo-bulk samples to empirically validate ATSDP-NET and demonstrate its utility in a clinical context. Given the current limitations, our goal is to refine single-cell modeling techniques and contribute to the evolving understanding of tumor heterogeneity, while setting the stage for further research into clinical applications.

## 2 Materials and methods

### 2.1 Datasets

We evaluated our model using four publicly available single-cell RNA sequencing (scRNA-seq) datasets, each representing a distinct drug treatment and cancer context:

- **DATA1**: human oral squamous cell carcinoma (OSCC) cells treated with Cisplatin.
- **DATA2**: additional OSCC scRNA-seq data also treated with Cisplatin.
- **DATA3**: human prostate cancer cells treated with Docetaxel.
- **DATA4**: murine acute myeloid leukemia (AML) cells treated with I-BET-762.

For each dataset, scRNA-seq was conducted on cancer cells before the administration of drug treatment, enabling the capture of pre-treatment transcriptomic states. After drug treatment, each cell was assigned a binary response label (0 = resistant, 1 = sensitive) based on post-treatment viability assays. These labels, derived from experimental annotations in the original publications, serve as the ground truth for model training and evaluation.

To address class imbalance in the labeled datasets, we applied different sampling strategies: SMOTE was used for DATA1, and oversampling was used for DATA2, DATA3, and DATA4.

These datasets, with well-defined pre-treatment input features and post-treatment binary response labels, support single-cell-level supervised learning and enable reliable evaluation using metrics such as AUC, accuracy, and F1 score. The original drug response values in the GDSC and CCLE databases were reported as continuous variables such as IC50 or percent viability. For this study, we adopted binary drug response labels (0 = resistant, 1 = sensitive) derived from existing annotations in the datasets or binarized based on established thresholds as defined in prior studies (e.g., top/bottom quantiles of response distributions). These binary labels serve as ground truth for model training and evaluation.

### 2.2 Data collection and preprocessing

In our study design, scRNA-seq data is collected before applying any drug treatment. This allows us to capture the baseline transcriptional state of each cell. The same cell population is then subjected to drug treatment, after which cellular response is assessed. To determine which cells responded by dying, we rely on dataset-provided viability labels, which were derived by the original authors using post-treatment assays. Although we do not directly observe or trace individual cells over time, the annotations provided link each cell's pre-treatment transcriptome to its post-treatment viability outcome.

Comprehensive datasets from the GDSC and CCLE databases were used in this study. These datasets included drug response profiles for 1,280 cancer cell lines, 1,557 drugs, and 15,962 genes. Drug-sensitive cell lines were annotated with a binary label of 1, whereas drug-resistant cell lines were labeled as 0. Predictive model performance may be negatively impacted by the notable class imbalance in the training dataset caused by the varying distribution of sensitive and resistant cell lines across drug treatments.

Oversampling and the Synthetic Minority Oversampling Technique (SMOTE) (29) were two sampling strategies used to optimize the training dataset structure to lessen the problem of class imbalance. SMOTE used a more complex technique by creating synthetic samples inside the minority class's feature space, increasing the diversity and representativeness of the training data, whereas oversampling duplicated samples from the minority class to increase their representation.

The bulk RNA-Seq datasets used for pre-training were split into 80% training and 20% test sets. Similarly, the single-cell RNA-Seq datasets used for fine-tuning and testing were also divided with an 80/20 ratio. This consistent splitting ensures that both stages (pre-training and fine-tuning/testing) are clearly separated and reproducible.

To address class imbalance in the datasets, different oversampling techniques were applied. Specifically, SMOTE was used for DATA1, while standard oversampling was used for DATA2, DATA3, and DATA4. The decision to use SMOTE for DATA1 was based on the significant class imbalance in this dataset, where a large proportion of drug-resistant cells were underrepresented. SMOTE was chosen to generate synthetic samples for the minority class, allowing the model to learn more effectively from the drug-resistant cells. For the other datasets (DATA2–4), the class imbalance was less pronounced, so standard oversampling (duplicating minority class samples) was deemed sufficient.

While SMOTE helps to balance the dataset by generating synthetic samples, it can also introduce some risk of overfitting, especially if the synthetic samples do not accurately represent the minority class. To ensure that our model's performance improvements are robust, we plan to evaluate alternative balancing strategies (such as undersampling or different oversampling techniques) in future work, and will report these results for comparison.

To justify the selection of different sampling strategies across datasets, we conducted a comparative experiment to evaluate the impact of SMOTE and simple oversampling on model performance. SMOTE yielded significantly better results for DATA1, which had a severe class imbalance, while simple oversampling consistently outperformed SMOTE for DATA2, DATA3, and DATA4, where the imbalance was less pronounced. These results demonstrate that our tailored preprocessing design is robust and appropriate for the specific class distributions in each dataset. This evidence supports the rationale for adopting SMOTE for severely imbalanced data and standard oversampling for datasets with moderate imbalance. We believe this careful selection improves model generalizability and minimizes the risk of overfitting associated with inappropriate sampling techniques.

Additionally, the following preparation procedures were used to improve the dependability and quality of the single-cell RNA-seq data: (i) To remove noise and non-informative features, cells with <200 recognized genes—a sign of empty droplets—and genes discovered in fewer than three cells were eliminated. These steps are particularly important in single-cell RNA-seq data, as they help to eliminate low-quality cells that could negatively impact downstream analysis. (ii) To prevent confounding effects, cells with mitochondrial gene expression levels higher than 10% of total gene expression were eliminated. This step is commonly used in single-cell data to avoid the inclusion of stressed or dying cells, which may distort the overall analysis. (iii) To make subsequent studies easier, the count matrix was normalized using the total UMI counts per cell and then logarithmically transformed. Normalization is especially crucial in single-cell RNA-seq data to ensure that cell-to-cell comparisons are valid and not influenced by technical variations. All of these preprocessing steps were performed on scRNA-seq data collected prior to drug treatment, ensuring that the model learns from the true pretreatment transcriptional states.

In this study, we use both bulk RNA-seq data and single-cell RNA-seq data for drug response prediction. For the bulk RNA-seq data used in this study, we use data derived from established drug response databases such as GDSC and CCLE. These databases provide drug response labels for cancer cell lines, denoted as *Y*_*g*_, where each label indicates whether the cell line is drug-sensitive or drug-resistant based on post-treatment assays. For the single-cell RNA-seq data, we use experimental drug response annotations obtained through assays such as Annexin V staining. These provide binary labels for individual cells, denoted as *Y*_*s*_, representing whether each cell is drug-sensitive (1) or drug-resistant (0) after treatment.

Throughout the manuscript, we distinguish between bulk-level drug response labels (*Y*_*g*_) and single-cell-level drug response labels (*Y*_*s*_) to avoid any ambiguity. The main innovation of this study is the creation of the ATSDP-NET model, even though these sampling and preprocessing methods enhanced the dataset's quality and balance. In contrast to conventional techniques, ATSDP-NET incorporates a deep transfer learning framework with a multi-head attention mechanism to precisely extract features specific to single-cell data, allowing for extremely precise drug response predictions. The model's ability to predict drug sensitivity and resistance with more accuracy and interpretability is greatly increased by this focused approach, which successfully tackles the difficulties associated with single-cell feature extraction.

### 2.3 Overview of the ATSDP-NET architecture

**Figure 1:**
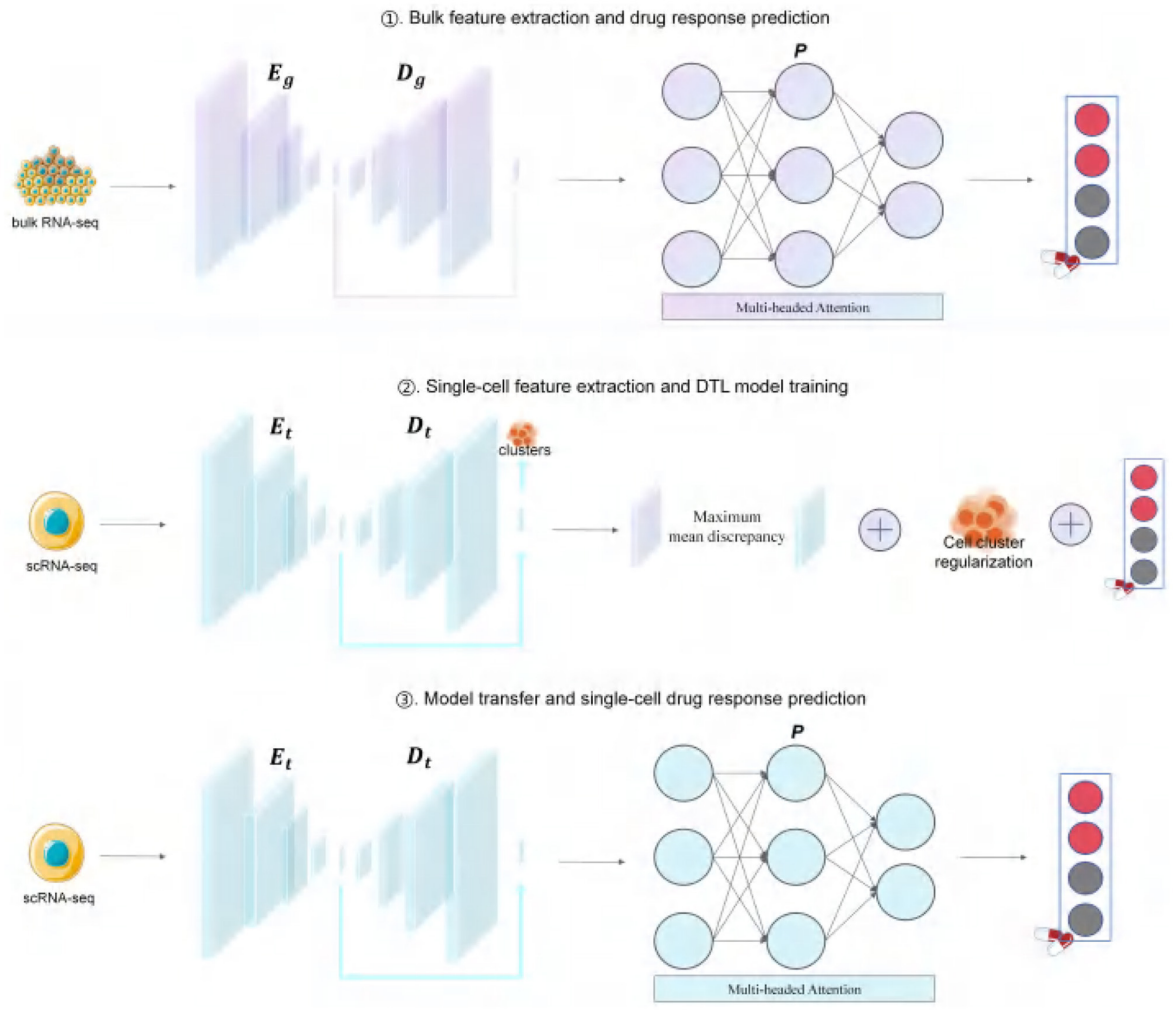
ATSDP-NET framework. RNA-seq data and corresponding drug response labels were obtained from the GDSC and CCLE databases and subsequently processed according to the ATSDP-NET framework. A denoising autoencoder (DAE) is employed to introduce noise into the bulk data, using an encoder (*E*_*g*_) and decoder (*D*_*g*_) to derive low-dimensional features. These low-dimensional feature matrices are then fed into a predictor (*P*) that integrates a multi-head attention mechanism and a multilayer perceptron to predict cell drug responses. A similar approach is applied to the single-cell data, where the DAE (comprising *E*_*t*_ and *D*_*t*_) is updated to extract the latent features of the single-cell data. The entire framework is trained by considering the maximum mean discrepancy (MMD) between the low-dimensional feature spaces of single-cell and bulk data, the cross-entropy loss between the predicted drug response values and the corresponding labels of bulk cells, and the regularization of the cell clusters predicted from scRNA-seq data. By minimizing the overall loss, *E*_*t*_, *D*_*t*_, and *P* are simultaneously updated and optimized.

Figure 1 summarizes the main pipeline of ATSDP-NET, which consists of three stages:

(i) An autoencoder is employed to extract gene expression features from bulk RNA-seq data, which are subsequently utilized by the predictor to perform drug response prediction.
(ii) The autoencoder is further leveraged to extract gene expression features from single-cell RNA-seq data, enabling joint training and model refinement.
(iii) Finally, the trained and transferred model is applied to single-cell RNA-seq data for accurate drug response prediction.

### 2.4 ATSDP-NET workflow design

The ATSDP-NET framework is structured into three distinct stages: (1) feature extraction from bulk RNA-seq data and drug response prediction, (2) feature extraction from single-cell RNA-seq data and deep transfer learning (DTL) model training, and (3) model transfer and single-cell drug response prediction.

#### 2.4.1 Feature extraction from bulk RNA-Seq data and drug response prediction

The first step of transfer learning involves utilizing a denoising autoencoder (DAE) to extract gene features from bulk RNA-seq data, reduce dimensionality, and denoise the data. Additionally, this step serves as a fine-tuned pretraining phase to initialize the predictor (P) weights for subsequent optimization in the second stage.

The DAE learns a low-dimensional representation of the bulk expression matrix *X*_*g*_, in which a gene is represented by each row and a cell line by each column. The DAE architecture comprises three main components:

1. **Noise injection (G):** this step generates a noisy version of the bulk expression matrix *X*_*g*_ by introducing random noise based on a binomial distribution, resulting in $X_{g}^{\prime }$:
  (1) $$\begin{array}{l} X_{g}^{\prime }=G\left(X_{g},F_{g}\right) \end{array}$$
  where *F*_*g*_ represents the probability of setting a value to zero in each row. The binomial distribution is used here because the data is assumed to be binary (e.g., gene expression is either “on” or “off,” representing active or inactive genes). The noise is injected as a binary response for each feature (gene), where each value has a probability *F*_*g*_ of being active (1) and a probability 1 − *F*_*g*_ of being inactive (0). This approach models the inherent variability in the data, simulating potential biological noise.
2. **Encoder (*E*_*g*_):** The encoder maps the noisy input *X'*_*g*_ into a lower-dimensional latent space with ReLU activation.
3. **Decoder (*D*_*g*_):** The decoder reconstructs the obtained low-dimensional embedding matrix into an approximate matrix *X”*_*g*_.

The DAE is optimized by minimizing the reconstruction loss function (mean squared error, MSE) between the input *X*_*g*_ and the reconstructed matrix *X”*_*g*_, ensuring that *X”*_*g*_ closely approximates *X*_*g*_. The training objective of the model can be expressed as:


(2) $$\begin{array}{l} \min{\mathrm{loss}}_{\text{recon}}\left(E_{g},D_{g},X_{g}\right)=\min\left(\mathrm{MSE}\left(X_{g},X_{g}^{\prime \prime }\right)\right) \end{array}$$

(3) $$\begin{array}{l} X_{g}^{\prime \prime }=D_{g}\left(E_{g}\left(X_{g}^{\prime }\right)\right) \end{array}$$

After completing the training of the DAE, drug response prediction for bulk cells is performed. Initially, a multi-head attention mechanism maps the input X through a linear transformation into three distinct spaces, generating the query vector Q, key vector K, and value vector V. For each attention head i, these vectors are dimensionally reduced, enabling the model to capture information across different representation subspaces. Specifically, for each head i:


(4) $$\begin{array}{l} Q_{i}=XW_{i}^{Q}, \end{array}$$

(5) $$\begin{array}{l} K_{i}=XW_{i}^{K}, \end{array}$$

(6) $$\begin{array}{l} V_{i}=XW_{i}^{V}, \end{array}$$

where $W_{i}^{Q}$, $W_{i}^{K}$, and $W_{i}^{V}$ represent the learnable weight matrices.

Subsequently, for each attention head, the attention scores are computed and the Softmax function is applied to obtain the attention weights:


(7) $$\begin{array}{l} \text{Attention}\left(Q,K,V\right)=\text{Softmax}\left(\frac{QK^{⊤}}{\sqrt{d_{k}}}\right)V \end{array}$$

where, *d*_*k*_ represents the dimensionality of the key vectors, and the scaling factor ($\sqrt{d_{k}}$) helps stabilize the training process.

Subsequently, the attention outputs from all heads are concatenated and passed through another linear transformation matrix *W*^*O*^ to generate the final multi-head attention output:


(8) $$\begin{array}{l} \text{MultiHead}\left(Q,K,V\right)=\text{Concat}\left({\text{head}}_{1},\ldots ,{\text{head}}_{h}\right)W^{O} \end{array}$$

(9) $$\begin{array}{l} {\text{head}}_{i}=\text{Attention}\left(Q_{i},K_{i},V_{i}\right) \end{array}$$

Finally, the predictor (*P*), which incorporates a multi-head attention mechanism and a fully connected multilayer perceptron (MLP), is trained on bulk RNA-seq data. The outputs from *P* are used to evaluate the relationship between drug responses and bulk gene expression profiles. The classification loss, specifically the cross-entropy between the predicted drug responses *Y*_*g*_ for each cell line and the binary drug response labels $Y_{g}^{\prime }$ extracted from the bulk database, is utilized to optimize the internal parameters of *P*:


(10) $$\begin{array}{l} \min{\text{loss}}_{\text{class}}\left(P,Y_{g},Y_{g}^{\prime }\right)=\min\left(\text{Cross Entropy}\left(Y_{g},Y_{g}^{\prime }\right)\right) \end{array}$$

(11) $$\begin{array}{l} Y_{g}=P\left(E_{g}\left(X_{g}^{\prime }\right)\right) \end{array}$$

#### 2.4.2 Single-cell feature extraction and DTL model training

In the second stage, feature extraction was performed on single-cell data by training a similar DAE model to extract features from scRNA-seq data *X*_*t*_, The loss function for this process can be formulated as:


(12) $$\begin{array}{l} \min{\text{loss}}_{\text{recon}}\left(E_{t},D_{t},X_{t}\right)=\min\left(\text{MSE}\left(X_{t},X_{t}^{\prime \prime }\right)\right) \end{array}$$

(13) $$\begin{array}{l} X_{t}^{\prime \prime }=D_{t}\left(E_{t}\left(X_{t}^{\prime }\right)\right) \end{array}$$

(14) $$\begin{array}{l} X_{t}^{\prime }=B\left(X_{t},p_{t}\right) \end{array}$$

where, $X_{t}^{\prime }$ introduces random noise with probability *p*_*t*_, inducing the noisy single-cell expression matrix. *E*_*t*_ and *D*_*t*_ denote the encoder and decoder, respectively, while $X_{t}^{\prime \prime }$ is the reconstructed scRNA-seq matrix produced by *D*_*t*_.

After training the DAE model for single-cell data, the domain transfer learning (DTL) model is subsequently trained. The DTL training leverages gene features extracted at both bulk and single-cell levels to predict cell sensitivity through the predictor *P*. The domain-adversarial neural network (DaNN) model incorporates an additional loss term called maximum mean discrepancy (MMD), which quantifies the similarity between the outputs of *E*_*g*_ and *E*_*t*_. MMD is defined as:


(15) $$\begin{array}{l} {\text{loss}}_{\text{MMD}}\left(E_{g}\left(X_{g}\right),E_{t}\left(X_{t}\right)\right)=|\frac{1}{n}\overset{n}{\underset{i=1}{\sum }}\phi \left(x_{g}^{i}\right)-\frac{1}{m}\overset{m}{\underset{j=1}{\sum }}\phi \left(x_{t}^{j}\right){|}_{H} \end{array}$$

Where $X_{g}={\left\{x_{g}^{i}\right\}}_{i=1,\ldots ,n}$ and $X_{t}={\left\{x_{t}^{j}\right\}}_{j=1,\ldots ,m}$ are data vectors from *n* bulk cell lines and *m* single cells, respectively; ϕ(·) denotes the mapping to the reproducing kernel Hilbert space (RKHS). The RKHS norm |·|_*H*_ measures the distance between vectors in different dimensions.

The similarity between gene features is incorporated into the classification loss during the training of the predictor *P*, ensuring that the feature spaces of *E*_*g*_ and *E*_*t*_ have similar distributions. The DaNN model is trained to update the two gene extractors *E*_*g*_ and *E*_*t*_ simultaneously, as well as the predictor *P*, and can be defined as:


(16) $$\begin{array}{l} \underset{E_{g},E_{t},P}{\min}{\text{loss}}_{\text{DaNN}}\left(X_{g},X_{t},E_{g},E_{t},P\right)={\text{loss}}_{\text{class}}\left(P,E_{g},X_{g},Y_{g}\right)+\\ \;\omega \cdot {\text{loss}}_{\text{MMD}}\left(E_{g}\left(X_{g}\right),E_{t}\left(X_{t}\right)\right)\\ \;+\theta \cdot \text{regularizer} \end{array}$$

(17) $$\begin{array}{l} \text{regularizer}=\underset{c\in CC}{\sum }{\text{cosine}}_{\text{similarity}}\left(c,X_{t}\right) \end{array}$$

where ω is the weight of the loss_MMD_(·), θ represents the weight of the regularization term, *c* denotes an individual cell, and *CC* corresponds to the clustering categories derived from the Louvain clustering results. By minimizing loss_DaNN_(·), the trained *E*_*t*_ and *P* will be used to predict drug responses in scRNA-seq data.

#### 2.4.3 Model transfer and single-cell drug response prediction

In the third stage, the model undergoes transfer learning to enable drug response prediction at the single-cell level. The primary objective is to leverage the feature extractor and predictor trained on bulk RNA-seq data and apply them to scRNA-seq data, thereby enabling the evaluation of drug response states in individual cells. The pre-trained feature extractor *E*_*t*_ is transferred to single-cell datasets and integrated with scRNA-seq data to construct a predictive framework for single-cell drug response.

Within this framework, the transferred *E*_*t*_ (feature extractor) and *P* (predictor) are jointly utilized to process scRNA-seq data as input. The feature extractor *E*_*t*_ performs dimensionality reduction and embedding operations on the high-dimensional gene expression data from single cells, extracting biologically meaningful latent features. Subsequently, *P* uses these embedded characteristics to generate drug response probability scores *Y*_*s*_ for each cell. These scores are represented as continuous probability distributions ranging from 0 to 1, providing a quantitative assessment of each cell's drug response state to a specific therapeutic agent. These continuous probability scores are used directly for model evaluation, including metrics such as AUC and Average Precision.

For downstream interpretation and analysis (e.g., identification of sensitive vs. resistant populations), we apply a post hoc binarization of these scores using a threshold of 0.5: cells with *Y*_*s*_ ∈ [0, 0.5] are classified as drug-resistant, while cells with *Y*_*s*_ ∈ [0.5, 1] are classified as drug-sensitive. This step is applied only after the prediction of the model and does not influence the training of the model or the evaluation of the performance.

Finally, to transform the continuous probability scores *Y*_*s*_ into interpretable binary labels, cells are categorized into two groups: cells with *Y*_*s*_ between 0 and 0.5 are defined as drug-resistant, while cells with *Y*_*s*_ between 0.5 and 1 are classified as drug-sensitive. This binarization is applied only for downstream analysis, such as visualization or gene marker identification. It is not part of the training process and does not affect the computation of evaluation metrics such as AUC or average precision.

This approach not only enables the model to predict each cell's drug sensitivity but also captures the heterogeneity of drug responses, offering a high-resolution perspective for investigating drug effects across cell populations.

#### 2.4.4 Evaluation metrics

Since each dataset includes experimentally annotated drug response outcomes at the single-cell level (i.e., binary ground-truth labels indicating whether each cell is drug-sensitive or drug-resistant), we formulate drug response prediction as a supervised binary classification task. These labels enable the use of standard evaluation metrics that assess how accurately the model predicts drug sensitivity.

We evaluate the performance of the ATSDP-NET model using a suite of well-established metrics: Precision, Recall, F1-Score, Area Under the Receiver Operating Characteristic Curve (AUC-ROC), and Average Precision (AP). These metrics offer complementary perspectives on classification performance:

**Precision** quantifies the proportion of true positives (TP) among all predicted positives. **Recall** measures the proportion of true positives correctly identified among all actual positives. **F1-Score**, the harmonic mean of Precision and Recall, balances their trade-off. **AUC-ROC** assesses the model's ability to distinguish between the two classes across different thresholds. **AP** captures the area under the Precision–Recall curve by computing the weighted mean of precision values across recall levels.

The following equations define the metrics used:


(18) $$\begin{array}{l} \text{Precision}=\frac{TP}{TP+FP} \end{array}$$

(19) $$\begin{array}{l} \text{Recall}=\frac{TP}{TP+FN} \end{array}$$

(20) $$\begin{array}{l} F1\text{–score}=\frac{2TP}{2TP+FP+FN} \end{array}$$

(21) $$\begin{array}{l} AP=\overset{n}{\underset{k=1}{\sum }}\left(R_{k}-R_{k-1}\right)P_{k} \end{array}$$

where *P*_*k*_ is the precision at the *k*-th threshold, *R*_*k*_ is the corresponding recall, and *n* is the number of thresholds considered. These metrics collectively enable a comprehensive evaluation of the model's ability to predict drug responses at the single-cell level.

## 3 Experiments and results

To evaluate the performance and effectiveness of the ATSDP-NET model, the following experimental setups were designed:

(i) Investigation of the impact of varying the number of attention heads in the multi-head attention mechanism on the performance of the ATSDP-NET model.

(ii) Comprehensive performance evaluation of the ATSDP-NET model in predicting single-cell drug responses.

(iii) Comparative testing of the ATSDP-NET model against various machine learning models to assess relative performance.

(iv) Analysis of drug response predictions in leukemia cells under I-BET treatment conditions.

(v) Identification of key genes associated with cisplatin drug response.

(vi) Validation of the strong correlation between drug response predictions and pseudotime analysis.

### 3.1 Experimental setup

The experimental environment was configured as follows: an 11th Gen Intel^®^ Core™ i7-11700HX CPU (2.50 GHz), NVIDIA GeForce GTX 1050 Ti GPU, 24 GB RAM, Windows 10 operating system, and PyTorch version 2.2.2.

The ATSDP-NET model was comprehensively evaluated on four publicly available single-cell RNA sequencing (scRNA-seq) datasets, each representing distinct drug treatments. The selected drugs include Cisplatin, I-BET-762, and Docetaxel, which are widely used in cancer therapy. Specifically, DATA1 and DATA2 comprise human oral squamous cell carcinoma (OSCC) cells treated with Cisplatin, DATA3 includes human prostate cancer cells treated with Docetaxel, and DATA4 involves murine acute myeloid leukemia (AML) cells treated with I-BET-762.

To address the class imbalance issue during training, SMOTE sampling was employed for the DATA1 dataset, while oversampling techniques were applied to the DATA2, DATA3, and DATA4 datasets. Each dataset contains binary drug response annotations for individual cells, where the labels were derived from the original data as binary indicators (0 for resistant cells and 1 for sensitive cells).

The hyperparameters of the ATSDP-NET model used in the experiments are summarized in Table 1, which outlines the configurations for each of the four datasets.

**Table 1:** Performance comparison of SMOTE and oversampling for handling class imbalance across datasets

**Dataset**	**Method**	**Precision**	**Recall**	**F1-score**	**AUC**	**AP**
Data1	SMOTE	0.8572	0.7786	0.7896	0.8578	0.9214
Data1	Oversampling	0.4947	0.4197	0.4293	0.4372	0.6235
Data2	SMOTE	0.4386	0.4929	0.3078	0.5123	0.7095
Data2	Oversampling	0.8468	0.7709	0.7811	0.8563	0.9274
Data3	SMOTE	0.7808	0.7654	0.7621	0.7661	0.7269
Data3	Oversampling	0.8327	0.8100	0.8058	0.9037	0.8952
Data4	SMOTE	0.3593	0.3594	0.3593	0.3109	0.5802
Data4	Oversampling	0.8726	0.8735	0.8740	0.8822	0.8456

### 3.2 Performance evaluation of ATSDP-NET for single-cell drug response prediction

To assess the performance of the ATSDP-NET model across different datasets, the model was tested on four datasets: DATA1, DATA2, DATA3, and DATA4. The evaluation aimed to measure the effectiveness of ATSDP-NET in predicting drug responses in various single-cell RNA sequencing (scRNA-seq) datasets under distinct experimental conditions.

**Figure 2:**
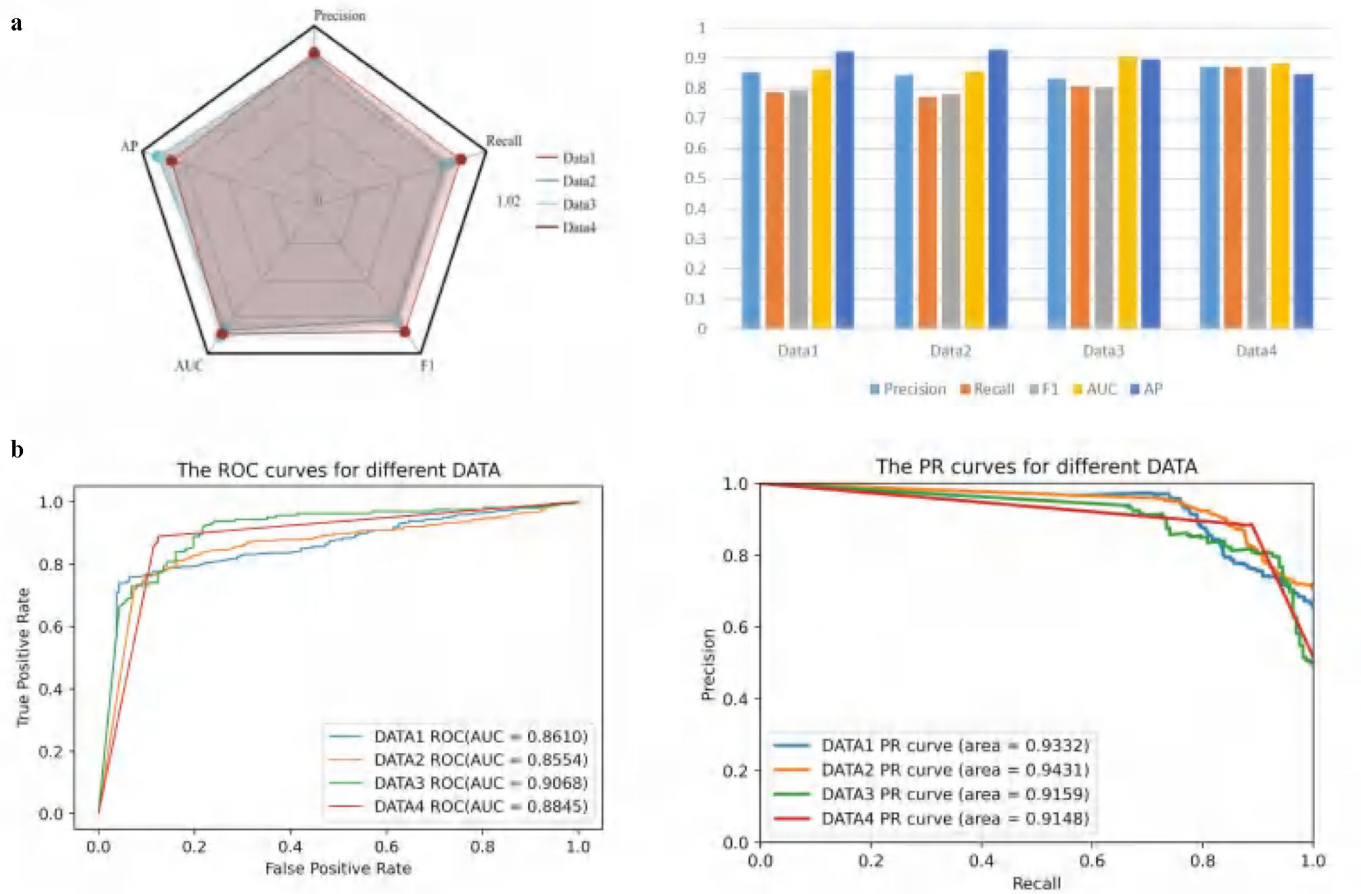
**(a, b)** Classification performance evaluation of ATSDP-NET model on four datasets. The figure includes radar charts, bar charts, ROC curves, and PR curves to evaluate the model's performance across different datasets. Each section represents a different evaluation metric for the four datasets.

Figure 2a consists of two subplots. The radar chart on the left illustrates the performance of the ATSDP-NET model across four datasets (DATA1, DATA2, DATA3, and DATA4) based on five key metrics: Precision, Recall, F1-Score, AUC, and AP. Each dataset is represented by a distinct color, allowing for an intuitive comparison of the model's performance across different datasets. The bar plot on the right presents the specific numerical values for the five performance metrics across each dataset. This visualization provides a clearer understanding of the detailed performance of the model on Precision, Recall, F1-Score, AUC, and AP, facilitating cross-dataset comparisons.

Figure 2b also consists of two subplots and is designed to showcase the classification performance of the model across the four datasets. The ROC curve on the left displays the Receiver Operating Characteristic (ROC) curves for each dataset along with their corresponding AUC values (Area Under the Curve). Higher AUC values indicate better classification performance. Notably, the AUC for DATA3 is the highest at 0.9068, demonstrating the best performance among the datasets. Meanwhile, DATA1, DATA2, and DATA4 also exhibit strong AUC values of 0.8610, 0.8554, and 0.8845, respectively.

The PR curve on the right visualizes the Precision-Recall (PR) curves and their corresponding areas for each dataset. The PR curve reflects the model's precision at varying levels of recall, making it particularly suitable for evaluating imbalanced datasets. It is evident that the PR curve area for DATA2 is the highest, reaching 0.9431, indicating superior performance on this dataset.

To evaluate the robustness of our method, we performed 10-fold stratified cross-validation on each dataset. To account for possible randomness due to data shuffling and model initialization, we repeated the entire training and evaluation process independently 10 times using different random seeds. The mean and standard deviation of performance metrics, including precision, recall, F1-score, AUC, and average precision, are reported in Table 2 to reflect the consistency and reliability of our results.

**Table 2:** Parameters of ATSDP-NET model on four datasets.

**Data**	**Bottleneck**	**Encoder dimensions**	**Predictor dimensions**	**Dropout**	**Epochs**
Data1 (GSE117872HN120)	512	256, 128	128, 64	0.3	500
Data2 (GSE117872HN137)	32	512, 256	256, 128	0.3	500
Data3 (GSE140440)	512	256, 128	256, 128	0.2	500
Data4 (GSE110894)	512	256, 128	128, 64	0.3	500

Table 3 summarizes the five key performance metrics of the ATSDP-NET model across four datasets, including Precision, Recall, F1-score, AUC, and AP, providing a comprehensive evaluation of the model's classification performance across different tasks. Overall, the ATSDP-NET model demonstrates high accuracy and stability across all metrics, highlighting its robustness and adaptability.

**Table 3:** ATSDP-NET results (mean ± std) under 10-fold CV with 10 runs.

**Dataset**	**Precision**	**Recall**	**F1**	**AUC**	**AP**
Data1	0.8572 ± 0.0069	0.7786 ± 0.0072	0.7896 ± 0.0077	0.8578 ± 0.0109	0.9214 ± 0.0085
Data2	0.8468 ± 0.0067	0.7709 ± 0.0084	0.7811 ± 0.0129	0.8563 ± 0.0070	0.9274 ± 0.0062
Data3	0.8327 ± 0.0082	0.8100 ± 0.0114	0.8058 ± 0.0110	0.9037 ± 0.0095	0.8952 ± 0.0093
Data4	0.8726 ± 0.0081	0.8735 ± 0.0092	0.8740 ± 0.0103	0.8822 ± 0.0076	0.8456 ± 0.0067

The results indicate that the model achieves a well-balanced performance between Precision and Recall, suggesting its ability to maintain predictive accuracy while effectively identifying positive samples. The F1-score further underscores the model's ability to harmonize precision and recall, showcasing superior classification performance on certain datasets. The AUC metric reflects the model's strong discriminatory power in distinguishing between positive and negative samples, while the AP metric validates the high-quality confidence scores of the predictions.

In conclusion, the results presented in Table 3 demonstrate that the ATSDP-NET model exhibits stable and outstanding performance across various datasets. It effectively addresses diverse data distributions and feature patterns, demonstrating strong generalization capabilities and classification performance.

### 3.3 Impact of different attention head counts on ATSDP-NET performance

**Figure 3:**
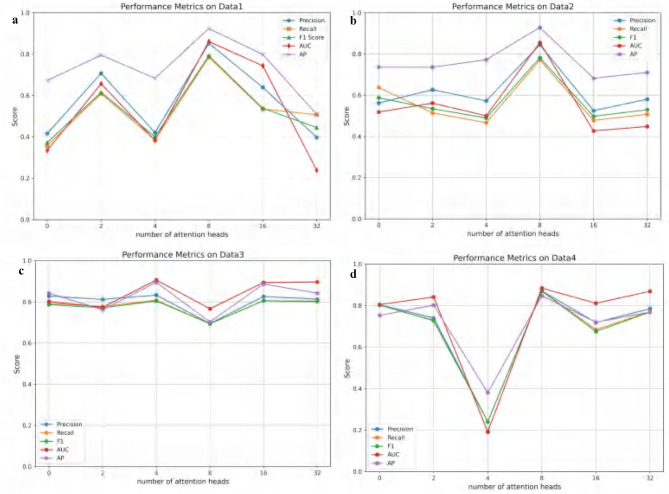
**(a–d)** Performance changes of ATSDP-NET model on four datasets under different numbers of attention heads (0, 2, 4, 8, 16, 32). The figure shows the performance variations as the number of attention heads increases, with each section labeled according to the corresponding number of attention heads.

In this section, the impact of varying attention head counts in the multi-head attention mechanism on the performance of the ATSDP-NET model is analyzed. Experiments were conducted on four datasets (DATA1, DATA2, DATA3, and DATA4), with the number of attention heads ranging from 0 to 32 within the ATSDP-NET framework. To comprehensively assess this influence, key performance metrics, including Precision, Recall, F1-score, AUC, and Average Precision (AP), were recorded under different attention head configurations. The trends in these performance metrics as a function of the number of attention heads are visualized in Figure 3, providing insights into the optimal configuration of the multi-head attention mechanism.

Figure 3 illustrates the impact of varying attention head counts on the performance of the ATSDP-NET model across four datasets. For DATA1 and DATA2, the model achieved optimal performance when the number of attention heads was set to 8. Under these conditions, the Precision, Recall, F1-score, AUC, and AP for DATA1 reached 0.8527, 0.7865, 0.7918, 0.8609, and 0.9239, respectively. Similarly, for DATA2, these metrics were 0.8448, 0.7711, 0.7816, 0.8553, and 0.9289, respectively. At this configuration, the ATSDP-NET model exhibited superior performance on both the PR and ROC curves. For DATA3, the optimal performance was observed when the number of attention heads was set to 4, with the highest Precision, Recall, F1-score, AUC, and AP recorded as 0.8332, 0.8086, 0.8051, 0.9067, and 0.8958, respectively. In DATA4, the model performed best with 8 attention heads, achieving Precision, Recall, F1-score, AUC, and AP values of 0.8711, 0.8703, 0.8704, 0.8845, and 0.8475, respectively. At these configurations, the model also achieved its peak performance on both the PR and ROC curves.

These experimental results highlight that 4 attention heads yielded the best performance for DATA3, while 8 attention heads significantly enhanced the accuracy and performance of the ATSDP-NET model for DATA1, DATA2, and DATA4. The findings underscore the critical importance of appropriately configuring the number of attention heads in multi-head attention mechanisms. A well-chosen head count can effectively improve the model's ability to capture and process information, providing valuable insights into the application of multi-head attention mechanisms for addressing complex data challenges.

### 3.4 Performance comparison across different machine learning models

In this study, we employed several deep learning methods, including Sparse Autoencoders (SA), Graph Attention Networks (GAT), Residual Networks (ResNet18), Transformers, Graph Convolutional Networks (GCN), and Convolutional Neural Networks (CNN), as well as two state-of-the-art drug response prediction models, DeepDR and DrugCell. These models were adapted to single-cell RNA-seq data to serve as baseline comparisons for our proposed ATSDP-NET. The performance of all models was evaluated using key metrics, including Precision, Recall, F1-score, Area Under the Curve (AUC), and Average Precision (AP).

The SA model is traditionally an unsupervised method for dimensionality reduction. To adapt it for drug response prediction, we used the autoencoder to extract sparse representations of the gene expression data, which were then passed through a classification layer. While SA is not inherently designed for classification tasks, its effectiveness for biological feature extraction has been demonstrated, for example, in Lei et al. (30). Including SA as a baseline highlights the advantage of supervised learning in our ATSDP-NET.

The ResNet18 and CNN models were originally designed for image data but were adapted here by reshaping the gene expression matrix into 1D vectors. This allowed us to leverage their feature extraction capabilities for single-cell gene expression profiles. We added fully connected layers for the final drug response prediction. Their potential for this task is supported by previous work, such as Chen and Jiang (31). Although these architectures are not optimized for RNA-seq data, they provide meaningful baselines for assessing whether ATSDP-NET, which is tailored for single-cell data, can outperform general-purpose models.

The Transformer model was employed without positional encoding to simplify the model and test the effectiveness of its self-attention mechanism on unordered gene expression data. Despite the absence of positional encoding, the Transformer could capture global dependencies among genes, as shown in Jiang et al. (32). This setup allowed us to examine whether a streamlined Transformer remains competitive for drug response prediction on single-cell data.

For GCN and GAT, we constructed graphs where each node represents a cell and edges reflect similarity between cells based on their gene expression profiles. An adjacency matrix was generated by applying a similarity threshold (e.g., 0.8) to determine connections. This graph-based representation enables the models to capture local relationships and community structures, enhancing predictive power. The effectiveness of this approach is supported by Yang et al. (33).

DeepDR and DrugCell are state-of-the-art drug response prediction models designed for bulk RNA-seq or multi-omics data. We adapted these models to our single-cell RNA-seq datasets for a comprehensive comparison. All baseline models were fine-tuned and evaluated using the same training, validation, and testing splits as ATSDP-NET to ensure a fair and consistent comparison.

To ensure reproducibility, each model was trained under the same conditions, with consistent hyperparameter tuning and evaluation protocols. The detailed implementation settings and parameter configurations are provided in the Supplementary material.

By adapting and fine-tuning these baseline models, we aimed to demonstrate the advantages of our proposed ATSDP-NET, which is specifically designed for single-cell drug response prediction.

The choice of deep learning methods over traditional baseline approaches, such as PCA+SVM, is motivated by the significant differences in data structure and processing requirements between single-cell RNA-seq data and bulk cell data (e.g., bulk RNA-seq data). Single-cell RNA-seq data typically exhibits high dimensionality and sparsity, making it difficult for traditional methods to effectively handle these characteristics, especially when integrating single-cell and bulk cell data. Deep learning methods, however, can automatically learn feature representations and effectively capture complex non-linear relationships within the data, making them more suitable for such complex datasets. Unlike traditional dimensionality reduction techniques like PCA, deep learning models do not rely on manual feature selection but instead use hierarchical learning mechanisms to automatically extract and integrate important features, thereby significantly improving the accuracy of drug response predictions.

Additionally, deep learning methods possess stronger generalization capabilities, enabling them to handle higher-dimensional and more complex patterns. In single-cell drug response prediction tasks, deep learning effectively integrates various types of data and, through multi-layered neural network learning, captures subtle cellular differences and potential non-linear patterns, further enhancing prediction accuracy. Therefore, the selection of deep learning methods is driven by the need to better handle the integration of single-cell and bulk cell data, while maximizing the accuracy of drug response predictions.

**Figure 4:**
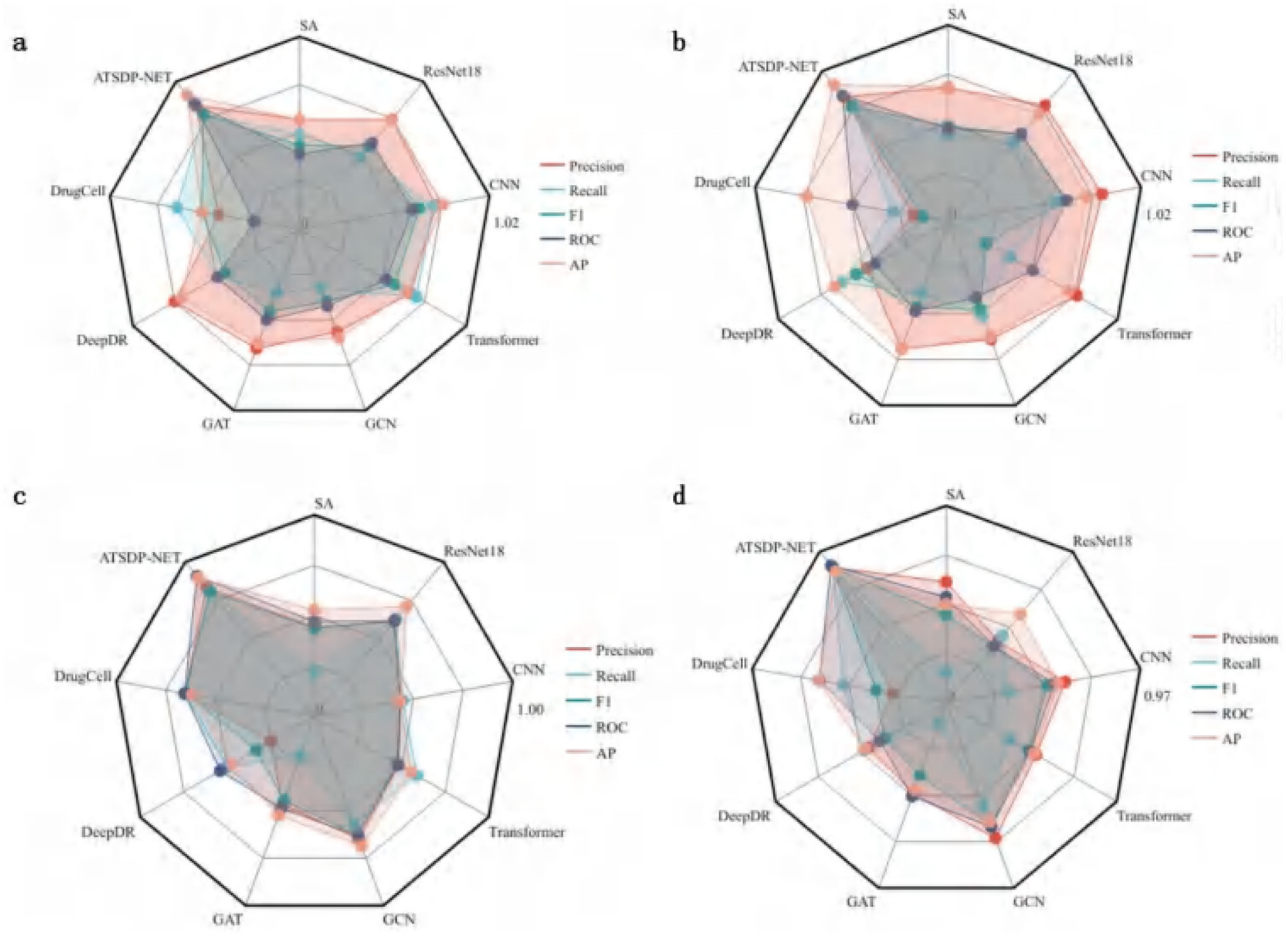
**(a–d)** Performance comparison of ATSDP-NET model and multiple models (SA, ResNet18, CNN, Transformer, GCN, GAT, DeepDR, DrugCell) on four datasets. The figure shows the performance of each model on the datasets, with part labels indicating the model names and corresponding results. ATSDP-NET, as the proposed method, is compared with existing models on all four datasets.

Figure 4a (DATA1) illustrates the differences in five key performance metrics across various models for the DATA1 dataset. The radar chart demonstrates that ATSDP-NET excels in Precision, Recall, AUC, and AP, highlighting its exceptional classification capability for the DATA1 dataset. In contrast, other models, such as SA and ResNet18, exhibit relatively poor performance, particularly in Recall and Precision. Models like DrugCell and DeepDR also show inferior performance compared to ATSDP-NET.

Figure 4b (DATA2) presents the evaluation results for the DATA2 dataset. ATSDP-NET continues to outperform other models in most metrics, with particularly notable advantages in Precision and F1-score. Although models such as Transformer and CNN show competitive performance in some individual metrics, ATSDP-NET consistently outperforms them across all key metrics in the DATA2 dataset, demonstrating its robustness and effectiveness in handling different datasets.

Figure 4c (DATA3) compares the performance of various models on the DATA3 dataset. While the performance differences between models are relatively small, ATSDP-NET still demonstrates a slight advantage in key metrics such as Precision, Recall, and F1-score, further validating its robust classification ability even when the performance differences across models are minimal.

Figure 4d (DATA4) presents the model performance on the DATA4 dataset. ATSDP-NET achieves near-optimal results across all five performance metrics, with particular superiority in Precision and AUC. In contrast, other models, such as GCN and CNN, show significantly lower performance in all metrics, while ResNet18 also lags behind ATSDP-NET.

Table 4 provides a clear comparison of the comprehensive superiority of ATSDP-NET across the five key metrics: Precision, Recall, F1-score, AUC, and AP. Notably, ATSDP-NET shows significant advantages across all metrics in both the DATA1 and DATA4 datasets, emphasizing its adaptability to drug response prediction tasks. This performance superiority is attributed to the multi-head attention mechanism embedded within the model, which effectively captures both global and local features in the data, thereby improving feature extraction and classification accuracy.

**Table 4:** Experimental results of ATSDP-NET model and multiple models on four datasets.

**DATA**	**SA**	**ResNet18**	**CNN**	**Transformer**	**GCN**	**GAT**	**DeepDR**	**DrugCell**	**ATSDP-NET**
**DATA1**									
Precision	0.5782	0.7563	0.7308	0.6763	0.5748	0.6683	0.7618	0.4339	**0.8527**
Recall	0.5013	0.4981	0.7146	0.7119	0.3296	0.3573	0.4982	0.6587	**0.7865**
F1	0.4427	0.5731	0.6404	0.5799	0.4039	0.4616	0.4567	0.5232	**0.7918**
ROC	0.3977	0.5944	0.6033	0.5271	0.4295	0.5075	0.5032	0.2432	**0.8609**
AP	0.5762	0.7522	0.7661	0.6572	0.6088	0.6398	0.7223	0.5242	**0.9239**
**DATA2**									
Precision	0.6958	0.7877	0.8162	0.7751	0.6543	0.7086	0.4873	0.1843	**0.8448**
Recall	0.4541	0.5434	0.5732	0.3769	0.5261	0.4044	0.6373	0.2904	**0.7711**
F1	0.4949	0.5916	0.6237	0.2301	0.4891	0.4785	0.5523	0.1308	**0.7816**
ROC	0.4846	0.5929	0.6291	0.5118	0.4236	0.4992	0.4404	0.5062	**0.8553**
AP	0.7001	0.7352	0.7335	0.7272	0.6422	0.7083	0.6843	0.7481	**0.9289**
**DATA3**									
Precision	0.4405	0.6144	0.4337	0.4822	0.6478	0.4657	0.2501	0.6515	**0.8332**
Recall	0.2283	0.6296	0.4444	0.5864	0.5679	0.2089	0.5001	0.6512	**0.8086**
F1	0.4364	0.6172	0.4321	0.4722	0.6281	0.4425	0.3334	0.6511	**0.8051**
ROC	0.4691	0.6172	0.4321	0.4783	0.6296	0.4845	0.5393	0.6539	**0.9067**
AP	0.5262	0.7093	0.4314	0.5534	0.6833	0.5214	0.4721	0.6169	**0.8958**
**DATA4**									
Precision	0.5971	0.3982	0.5973	0.5127	0.7112	0.4762	0.4379	0.2673	**0.8711**
Recall	0.1542	0.4324	0.3111	0.3637	0.5383	0.1071	0.4693	0.5165	**0.8703**
F1	0.4341	0.3651	0.5094	0.4692	0.6442	0.3811	0.3511	0.3523	**0.8704**
ROC	0.5215	0.3656	0.5429	0.4804	0.6516	0.4916	0.3776	0.6363	**0.8845**
AP	0.4874	0.5713	0.5594	0.5017	0.6244	0.4574	0.4624	0.6411	**0.8475**

The bold values represent the best performance for each metric across datasets, indicating the highest achieved results for precision, recall, F1 score, ROC, and AP.

In contrast, other models exhibit certain limitations. For instance, while ResNet18 and Transformer show competitive performance in some metrics, they fall short in Recall and F1-score, reflecting their inability to capture the deep features inherent in single-cell data. Traditional deep learning models, such as GCN and CNN, lack specific design optimizations for drug response prediction tasks and therefore fail to deliver satisfactory performance across all datasets.

In conclusion, compared to the eight other models (SA, GAT, ResNet18, Transformer, GCN, CNN, DrugCell, and DeepDR), ATSDP-NET consistently demonstrates superior performance across all four datasets (DATA1, DATA2, DATA3, DATA4). These results underscore the critical role of the multi-head attention mechanism in enhancing the model's robustness and adaptability, enabling ATSDP-NET to more effectively capture significant features within the data, thereby improving classification performance.

### 3.5 Drug response prediction analysis in leukemia cells treated with I-BET

**Figure 5:**
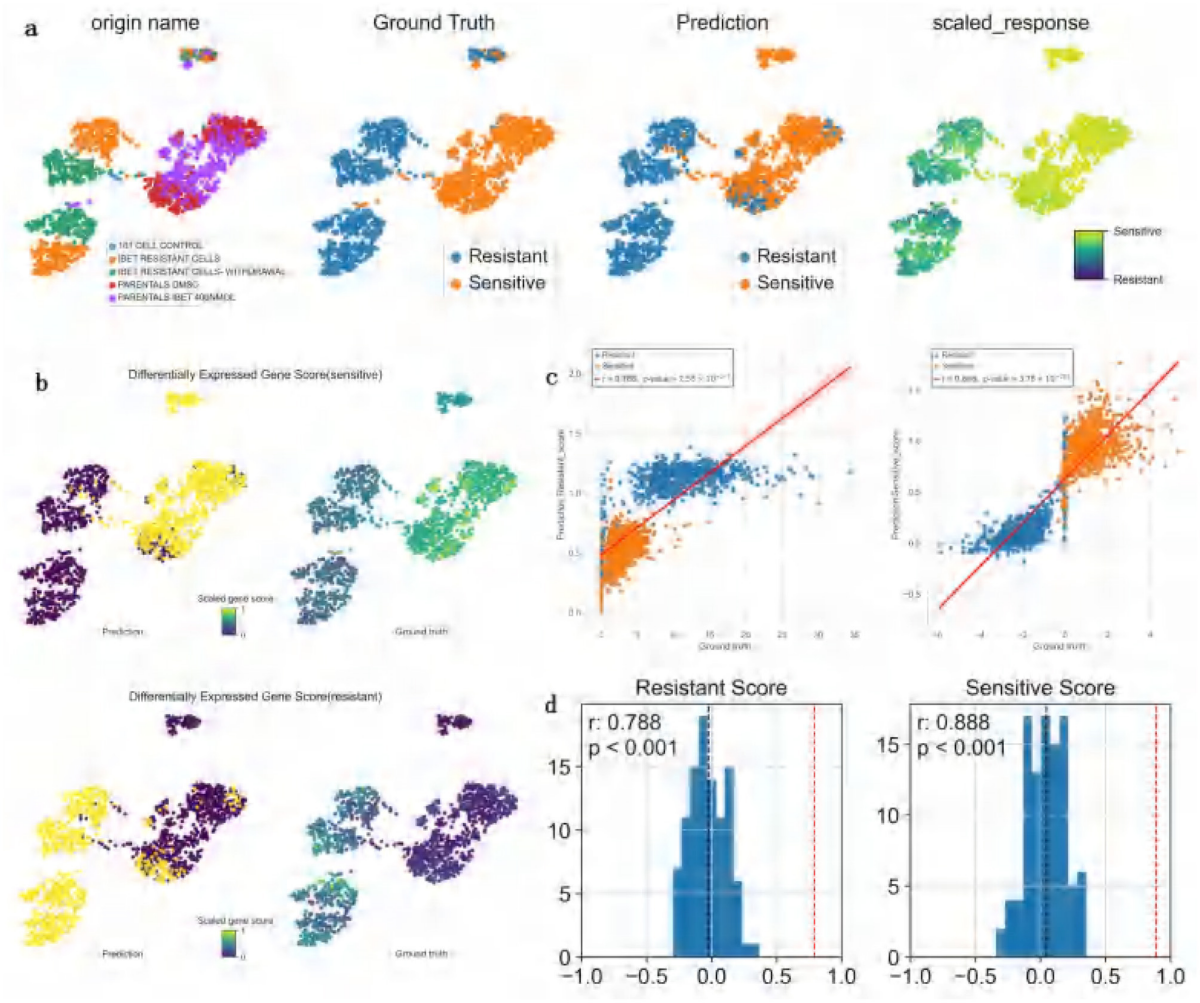
Visualization of the impact of I-BET treatment on dataset 4. **(a)** UMAP visualization of Dataset 4. Each column represents a distinct coloring scheme based on specific information: the first column is colored by treatment type as defined in the original study (e.g., “101 CELL CONTROL,” “BET RESISTANT CELLS,” etc.); the second column is colored by the ground-truth drug response labels (sensitive or resistant); the third column displays the binary drug response labels predicted by the model (sensitive or resistant); and the fourth column illustrates the predicted continuous drug response probability scores, transitioning from sensitive to resistant using a gradient-based color scheme. **(b)** UMAP visualization of gene scores. The top plot depicts the sensitive gene scores, while the bottom plot shows the resistant gene scores. These visualizations highlight the sensitive (or resistant) gene scores for differentially expressed genes within predicted and true clusters of sensitive (or resistant) cells. The intensity of the scores is represented using a color gradient (ranging from high to low). **(c)** Pearson correlation analysis between predicted and true gene scores. The left plot represents resistant gene scores, and the right plot represents sensitive gene scores. Scatterplots display the relationship between predicted and true gene scores, with red regression lines illustrating the degree of fit between predictions and ground truth. **(d)** Correlation analysis of resistant and sensitive gene scores. The left plot shows the distribution of correlation coefficients for resistant gene scores, while the right plot shows the distribution for sensitive gene scores. The x-axis represents the correlation of differentially expressed gene scores, and the y-axis represents frequency. The red dashed line indicates the correlation coefficient achieved by ATSDP-NET.

In the study of DATA4 (GSE110894), ATSDP-NET was employed to predict the drug response efficacy of a BET inhibitor (I-BET) in 1,419 leukemia cells characterized by AF9 gene fusions (Figure 5a). The experimental design consisted of five distinct conditions: one control group without any specific treatment (101 CELL CONTROL), two drug sensitivity states (DMSO and I-BET 400 NMOL), and two drug resistance states (I-BET resistance and I-BET withdrawal). Notably, the I-BET withdrawal group refers to cells in which drug treatment was discontinued at a specific time point to evaluate cellular responses or state changes post-withdrawal. Comparative analysis with the original study data demonstrated that ATSDP-NET achieved highly consistent results in predicting drug responses.

ATSDP-NET provided two approaches for drug response prediction: a continuous probability score and binary sensitivity/resistance labels. The continuous probability score reflects the likelihood of a cell's sensitivity to the drug, with higher scores indicating greater sensitivity. The binary labels are derived by applying a threshold to the continuous probability scores: cells with scores ranging from 0 to 0.5 are categorized as resistant, whereas those with scores from 0.5 to 1 are classified as sensitive.

In addition, gene scores were utilized to reflect the overall expression levels of differentially expressed genes (DEGs) within clusters of sensitive or resistant cells. The underlying assumption of this gene scoring approach is that accurate predictions should assign the correct drug response labels to individual cells. Consequently, the DEG scores for resistant and sensitive states should exhibit consistent correlations with DEGs derived from the ground truth data.

The analysis revealed that the generated DEG scores outperformed the DEG scores derived from ground truth labels in distinguishing resistant and sensitive cells (Figure 5b). Specifically, in the resistant DEG list, the correlation between the predicted and true DEG scores reached *R*^2^ = 0.788, while in the sensitive DEG list, the correlation was *R*^2^ = 0.888 (Figure 5c).

To assess the statistical significance of these correlations, 1,000 random tests were performed by selecting random genes in equal numbers to the predicted DEG list, and the correlations were recalculated. The results demonstrated that, across 1,000 random trials, the p-values for the correlations of resistant and sensitive DEG scores were both < 0.001, indicating that the observed correlations were statistically significant and robust (Figure 5d).

### 3.6 Identification of key genes associated with cisplatin drug response

Although ATSDP-NET demonstrates high accuracy in predicting single-cell drug responses, its greater value lies in its ability to uncover the key molecular mechanisms underlying drug resistance (34). In this study, ATSDP-NET was employed to analyze single-cell data from oral squamous cell carcinoma (OSCC) cells treated with cisplatin, aiming to identify critical gene markers associated with cisplatin resistance (35) and assess their potential clinical applications.

Cisplatin, a widely used chemotherapeutic agent, primarily induces cancer cell apoptosis by causing DNA damage (36). However, cancer cells often develop resistance to cisplatin by activating DNA repair pathways or inhibiting apoptosis (37). Through high-resolution single-cell analysis enabled by ATSDP-NET, several key genes (KGs) associated with cisplatin resistance were identified. Many of these genes were significantly enriched in resistant cell populations, suggesting their potential role as mediators of cisplatin resistance.

**Figure 6:**
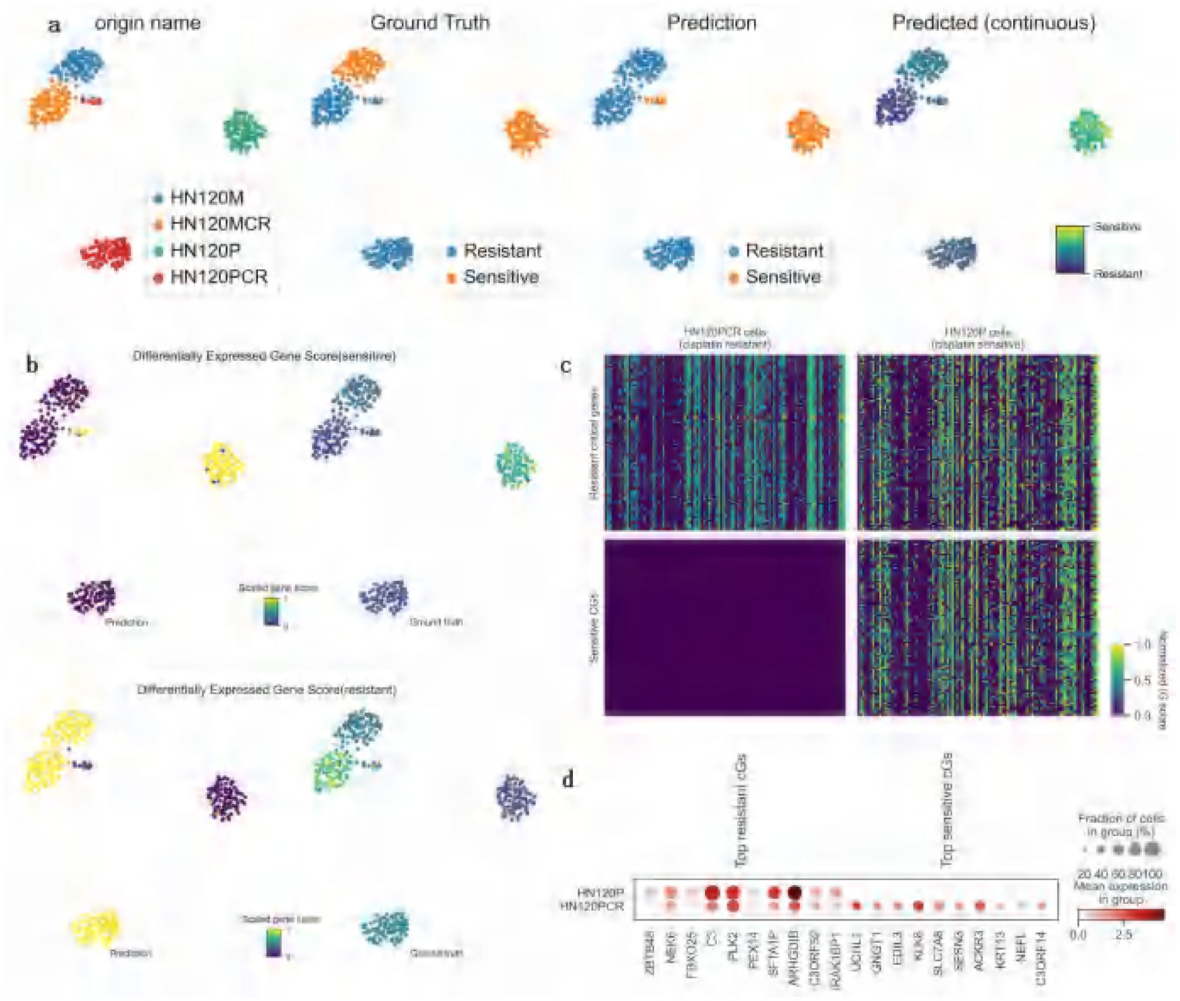
Case study on cisplatin Drug Response Analysis (DATA1). **(a)** Visualization of ATSDP-NET predictions for cisplatin drug response: the UMAP plots illustrate a comparison between the ground truth binary drug response labels and the ATSDP-NET-predicted binary drug response labels. Cells are categorized by their origin: blue represents HN120M cells, green represents HN120MCR cells, orange represents HN120P cells (drug-sensitive), and red represents HN120PCR cells (drug-resistant). The continuous response score plot on the right further highlights the probability of drug sensitivity for each cell, with the color gradient ranging from resistance (dark color) to sensitivity (light color), providing a detailed representation of the drug response levels across individual cells. **(b)** UMAP visualization of Differentially Expressed Gene (DEG) scores for sensitive and resistant cells: the top plot shows sensitivity gene scores, while the bottom plot displays resistance gene scores. DEG scores in the HN120P cell group (cisplatin-sensitive) and the HN120PCR cell group (cisplatin-resistant) are visualized using a color gradient (from low to high). The UMAP plots for both predicted results and ground truth labels demonstrate the model's capability in identifying sensitivity and resistance states among cells, indicating a strong agreement between predictions and actual states. **(c)** Integrated Gradient (IG) heatmap of the top 100 Key Genes (KGs) in HN120P and HN120PCR cell groups: the heatmap visualizes the expression levels of key genes, with each cell representing the corresponding gene expression value. Key genes highly expressed in the HN120PCR group are identified as associated with drug resistance, while those in the HN120P group are linked to drug sensitivity. The distinct gene expression patterns revealed in the heatmap provide insights into the molecular mechanisms underlying cisplatin sensitivity and resistance. **(d)** Expression patterns and cell fractions of the Top 10 key genes in HN120P and HN120PCR cell groups: the scatterplots depict the expression characteristics of the top 10 key genes in both cellular states. The size of the circles represents the fraction of cells expressing each gene within the respective group, while the color indicates the normalized average expression level. The left panel highlights the top 10 key genes specifically expressed in resistant cells, while the right panel focuses on those uniquely expressed in sensitive cells. IG refers to integrated gradient scores; KGs denotes key genes.

In this study, ATSDP-NET was applied to analyze the gene expression profiles of cisplatin-sensitive and resistant cells, uncovering critical molecular mechanisms associated with drug resistance and sensitivity. Figure 6a illustrates the classification and prediction results across four distinct cell sources. The ATSDP-NET model effectively distinguished resistant and sensitive cell populations, with predictions highly consistent with ground truth labels, demonstrating robust classification performance and precise identification of drug responses. Furthermore, the predicted continuous response distribution revealed a gradient from resistance to sensitivity, highlighting the heterogeneous nature of drug sensitivity among cancer cells.

Figure 6b illustrates the differentially expressed gene (DEG) scores for sensitive and resistant cells. The high DEG scores observed in sensitive cells suggest that these genes may play critical roles in cisplatin-induced apoptosis. Conversely, the distribution of DEG scores in resistant cells indicates that these genes may be involved in key resistance mechanisms, such as enhanced DNA repair pathways (38) and apoptosis inhibition (39), which enable cells to counteract cisplatin toxicity effectively.

Figure 6c presents a heatmap depicting the expression patterns of key resistance and sensitivity genes, categorized into “resistance-associated key genes (40)” and “sensitivity-associated key genes (41).” In cisplatin-resistant cells (HN120PCR), resistance-associated key genes exhibit elevated expression, while cisplatin-sensitive cells (HN120P) predominantly display higher expression of sensitivity-associated key genes. This contrasting gene expression pattern suggests that resistant cells may upregulate resistance-associated key genes to bolster their ability to withstand cisplatin treatment. In contrast, sensitive cells appear to activate sensitivity-associated key genes to enhance their responsiveness to cisplatin.

Figure 6d provides the names and distribution of specific resistance- and sensitivity-associated key genes. For instance, resistance-related genes such as ZBTB48 (42), NEK6 (43), and FBXO25 (44) show elevated expression in resistant cell populations, potentially contributing to enhanced DNA repair and anti-apoptotic pathways, thereby strengthening cellular resistance to cisplatin. In contrast, sensitivity-related genes such as UCHL1 (45), GNGT1 (46), and EDIL3 (47) exhibit significant expression in sensitive cells, potentially promoting apoptosis and increasing cellular sensitivity to cisplatin.

In summary, the ATSDP-NET model successfully identified key gene groups associated with cisplatin sensitivity and resistance, revealing distinct gene expression patterns between resistant and sensitive cells. Resistant cells appear to upregulate resistance-associated key genes to enhance DNA repair and survival pathways, while sensitive cells activate sensitivity-associated key genes to expedite apoptosis triggered by cisplatin. These findings provide novel insights into the mechanisms underlying cisplatin resistance and sensitivity, offering a foundation for developing personalized therapeutic strategies aimed at targeting resistance-associated genes or activating sensitivity-related genes.

### 3.7 Correlation between predicted drug response and pseudotime analysis

**Figure 7:**
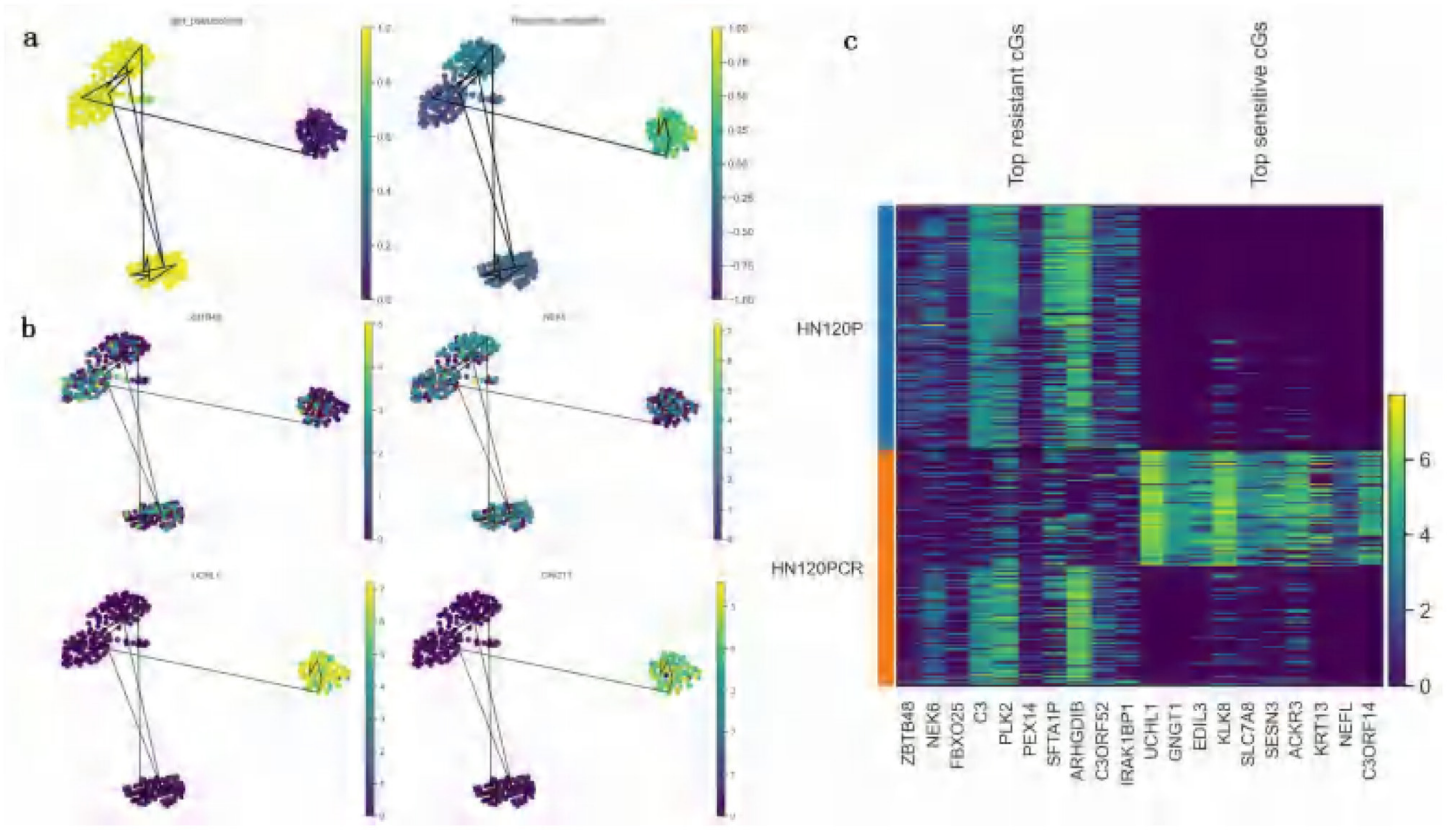
Validation of predicted drug responses based on pseudotime trajectory analysis (DATA1, cisplatin treatment). **(a)** UMAP visualization of pseudotime analysis and predicted drug response probabilities: the left panel depicts the UMAP visualization of pseudotime scores inferred from the original scRNA-seq data. Each cell is color-coded according to its pseudotime score, with a gradient ranging from yellow (early pseudotime) to purple (late pseudotime), reflecting the progression of cellular states during the cell cycle or differentiation process. The right panel illustrates the UMAP visualization of drug response probabilities predicted by ATSDP-NET. Cells are color-coded by their predicted drug sensitivity probabilities, with a continuous gradient ranging from deep purple (resistance) to yellow (sensitivity). The consistent gradient patterns between pseudotime scores and drug response probabilities suggest a strong association between pseudotime trajectories and cellular drug responses. **(b)** UMAP visualization of key gene expression: this panel highlights the expression patterns of representative key genes (KGs) in sensitive (bottom) and resistant (top) cell populations. In resistant cell populations, genes such as ZBTB48 and NEK6 are highly expressed, whereas genes like UCHL1 and GNGT1 show elevated expression in sensitive cell populations. These gene expression profiles provide molecular markers that characterize the drug response states of cells, offering insights into the underlying biological mechanisms of sensitivity and resistance. **(c)** Heatmap of key gene expression in sensitive and resistant cell populations: the heatmap displays the expression patterns of the top 10 key genes associated with resistance and the top 10 key genes associated with sensitivity in both cell populations. The color intensity represents the level of gene expression, where genes such as ZBTB48 and NEK6 exhibit high expression in resistant cell populations, while genes like UCHL1 and GNGT1 are predominantly expressed in sensitive cell populations. These distinct gene expression patterns provide crucial molecular signatures for identifying the drug response states of cells, facilitating a deeper understanding of the molecular mechanisms driving sensitivity and resistance to cisplatin treatment.

To validate the relationship between the predicted drug responses and the progression of drug treatment, trajectory inference was performed on DATA1 (treated with cisplatin). Pseudotime trajectories were inferred using the Diffusion Pseudotime (DPT) method implemented in Scanpy (48) in Python. Cells were pre-processed with PCA and UMAP for dimensionality reduction, a k-nearest neighbor graph was constructed, and diffusion maps were computed to estimate the pseudotime ordering. Pseudotime analysis revealed a trajectory where cells transitioned progressively from a sensitive state to a resistant state (Figure 7a). When comparing pseudotime results with the predicted drug response probability scores mapped onto the same diffusion UMAP, a clear trend was observed: as pseudotime progressed, the probability of resistance steadily increased, while sensitivity probability decreased correspondingly (Figure 7a).

These findings suggest that cells surviving high-dose drug treatment exhibited a marked drug-resistant phenotype, which aligns closely with the experimental (ground truth) labels of drug response states. This strong correlation highlights the predictive accuracy of ATSDP-NET in capturing the dynamic progression of drug responses at the single-cell level.

To further elucidate the molecular mechanisms underlying the development of drug resistance, the expression dynamics of key genes (KGs) identified by ATSDP-NET were analyzed along the pseudotime trajectory. Figure 7b highlights the expression patterns of two representative resistance-associated genes (ZBTB48 and NEK6) and two sensitivity-associated genes (UCHL1 and GNGT1). The expression levels of these genes align with the pseudotime trajectory trends and are consistent with the predicted drug response probability scores. Resistance-associated genes exhibit elevated expression during the later stages of pseudotime progression, whereas sensitivity-associated genes show higher expression during the early stages. This suggests that these genes play distinct regulatory roles in resistant and sensitive cellular states. It should be noted that pseudotime is an inferred ordering based on transcriptional similarity and does not incorporate actual treatment time or drug exposure information. Therefore, the observed increase in resistance probability may represent an association rather than a causal relationship.

Further analysis revealed that the predicted list of key genes (KGs) demonstrated significantly greater expression differences in distinguishing sensitive and resistant cell states compared to the differentially expressed genes (DEGs) (Figure 7c). This underscores the ability of ATSDP-NET to accurately identify molecular markers that are critical for characterizing drug response states and provides valuable insights into the regulatory mechanisms driving drug sensitivity and resistance.

**Figure 8:**
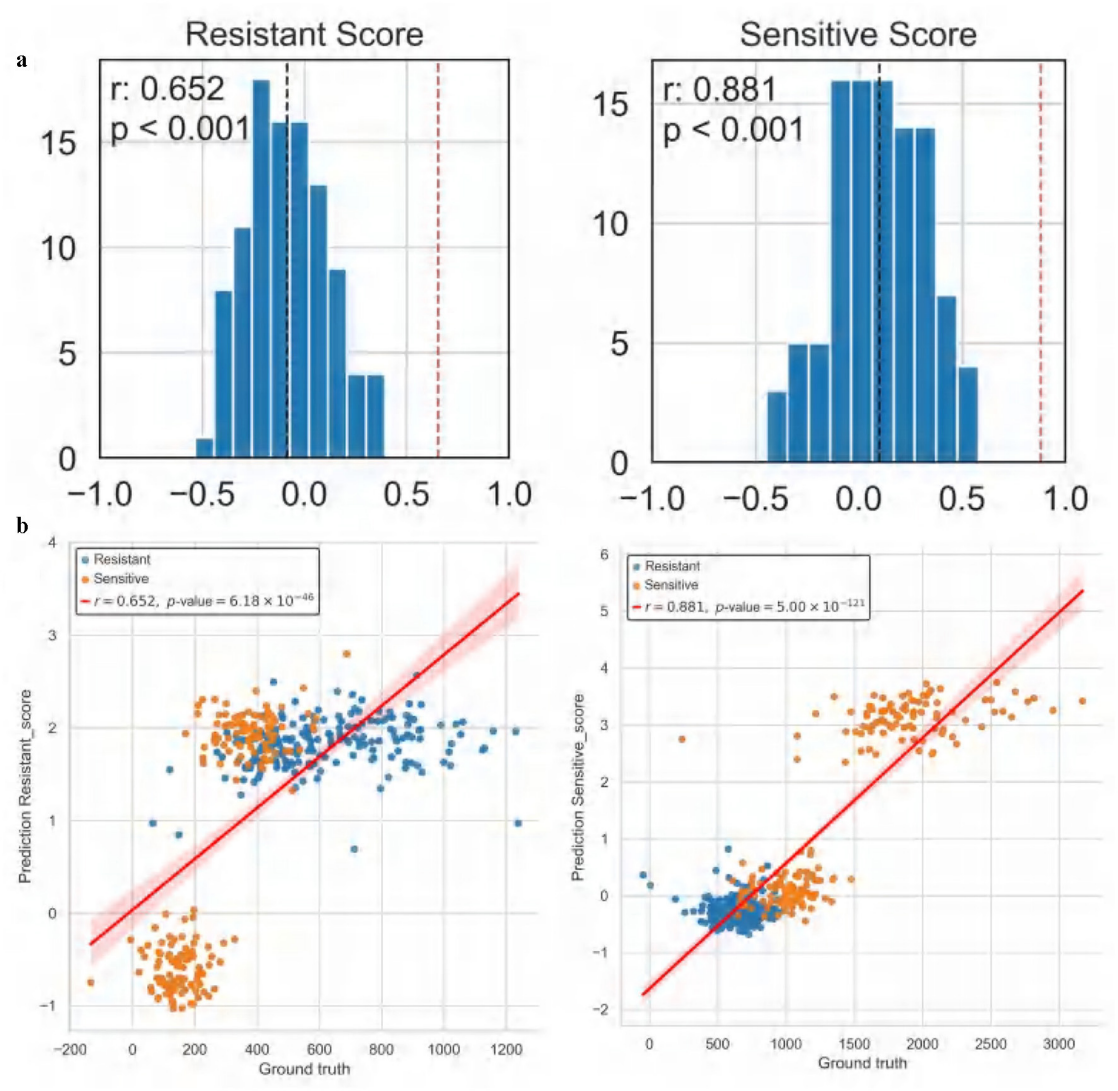
Correlation analysis of cisplatin drug response (Dataset 1). **(a)** Correlation analysis of resistance and sensitivity scores: the left panel presents the distribution of correlation coefficients for resistance-associated gene scores, while the right panel illustrates the distribution for sensitivity-associated gene scores. The X-axis denotes the correlation of differentially expressed gene (DEG) scores, and the Y-axis represents the frequency. The red dashed line indicates the correlation coefficient achieved by ATSDP-NET, demonstrating its predictive performance. **(b)** Pearson correlation between predicted and actual gene scores: the left panel shows the correlation between predicted and actual resistance-associated gene scores, while the right panel displays the correlation for sensitivity-associated gene scores. Scatter plots illustrate the relationship between predicted and actual gene scores, with red regression lines indicating the degree of fit. These results highlight the strong concordance between ATSDP-NET's predictions and the ground truth labels, further validating its capability to accurately capture key gene-level characteristics related to drug response.

The experimental results demonstrate that ATSDP-NET not only effectively predicts drug response probabilities but also uncovers the dynamic changes in cellular states through pseudotime analysis, providing a robust molecular basis for understanding the mechanisms underlying drug resistance. Furthermore, within the resistance-associated DEG list, the correlation between predicted DEG scores and actual DEG scores reached an R^2^ of 0.652, while the correlation for the sensitivity-associated DEG list achieved an R^2^ of 0.881 (Figure 8). These findings underscore the model's predictive capabilities, particularly its superior performance in identifying drug-sensitive cells. The significant correlation between sensitivity and resistance scores highlights the potential of ATSDP-NET as a reliable tool for drug response prediction in biomedical research.

## 4 Discussion

To further enhance the performance and scalability of ATSDP-NET, several improvement strategies are proposed in this study. The current version of ATSDP-NET has been optimized under a drug-specific training strategy, where the model is trained individually for each drug due to the distinct mechanisms of action and cellular responses associated with different drugs. However, we acknowledge that this strategy may limit the model's applicability in multi-drug scenarios. To address this limitation, we plan to explore multi-drug joint training in future research, which would improve the scalability of ATSDP-NET in handling multi-drug settings. Additionally, integrating larger, more diverse databases will enhance the model's robustness by increasing the diversity of training data.

Previous studies have demonstrated that transcriptional dynamics inferred from pseudotime can reflect the emergence of drug resistance under treatment pressure (49, 50) However, additional lineage tracing or time-series sampling would be needed to fully validate the biological trajectory suggested by our analysis.

One important aspect we are also focusing on is the interpretability and explainability of ATSDP-NET. To improve model explainability, we plan to incorporate attention visualization techniques that will allow researchers to understand which features the model deems most relevant for drug response prediction. By utilizing these techniques, we aim to provide more transparency in how the model makes its predictions, which is critical for clinical adoption. Furthermore, we will explore methods like layer-wise relevance propagation (LRP) to highlight the contributions of individual genes or features, thereby improving the model's interpretability and trustworthiness. This aspect will be crucial in facilitating the model's integration into clinical workflows and enhancing its practical utility.

Hyperparameter optimization through cross-validation and parameter tuning will also be employed, with a particular focus on refining the multi-head attention mechanism and other critical components of the model. Moreover, the application of advanced techniques such as transfer learning and domain adaptation will be explored to improve the generalization capability of ATSDP-NET on new datasets. Transfer learning, particularly for small-scale or feature-sparse datasets, will enable the model to leverage prior knowledge from annotated datasets, thereby boosting its performance in new tasks.

Regarding cross-species validation, we recognize that the experimental work presented in this study is based primarily on data from a single species. We plan to extend the model's applicability by validating its robustness and adaptability across multiple species and diverse conditions in future studies. The incorporation of multi-drug, multi-condition, and cross-species datasets will further strengthen the model's scalability and generalizability.

In addition, we aim to investigate the potential integration of ATSDP-NET with other machine learning frameworks, such as the use of generative models to simulate drug responses under various conditions. This approach would allow for a more comprehensive understanding of the factors influencing drug efficacy. Lastly, to facilitate the practical application of ATSDP-NET in clinical settings, we plan to prioritize the development of user-friendly tools and interfaces. This will support the advancement of precision medicine and accelerate ATSDP-NET's adoption by researchers and clinicians.

Although we report the mean and standard deviation of performance metrics across multiple runs to demonstrate robustness, we acknowledge that the current manuscript does not include performance distribution plots (e.g., boxplots) or statistical significance tests comparing ATSDP-NET with baseline models such as DrugCell. We recognize the value of such analyses and plan to incorporate comprehensive significance testing (e.g., *t*-tests) and visualizations in future work to further strengthen the credibility of our conclusions.

In this work, we have focused on developing the ATSDP-NET model for predicting drug responses at the single-cell level, incorporating deep transfer learning and multi-head attention mechanisms. We considered the approaches proposed by Fustero-Torre et al. (51) and Suphavilai et al. (52) for comparison, but we found that their methods could not be directly adapted to our problem in a way that would allow for a fair comparison.

The main reason for this is the different approaches these methods take in handling drug response prediction. Specifically: Fustero-Torre et al. focus on targeting cancer therapeutic heterogeneity using single-cell RNA-seq data. Their method addresses specific cancer therapeutic challenges and does not incorporate techniques like deep transfer learning or multi-head attention, which are central to our ATSDP-NET model. Suphavilai et al. predict heterogeneity in clone-specific therapeutic vulnerabilities using single-cell transcriptomic signatures. However, their approach focuses on predicting therapeutic vulnerabilities at a higher level of resolution and does not directly align with our drug response prediction task at the single-cell level.

Due to these fundamental differences in approach, we were unable to adapt their methods for a fair comparison. Our ATSDP-NET method, which leverages deep transfer learning and multi-head attention, enables accurate and interpretable predictions of drug responses at the single-cell level, an aspect not addressed by the methods proposed in these papers.

Therefore, while both of these papers present valuable methods for analyzing single-cell data, their approaches do not lend themselves to a direct comparison with our model. We believe that the unique aspects of ATSDP-NET provide a strong contribution to predicting drug responses at the single-cell level.

## 5 Conclusions

ATSDP-NET is a model specifically designed for the analysis of single-cell RNA sequencing (scRNA-seq) data, demonstrating significant potential in predicting drug responses for cancer and other diseases. In this study, ATSDP-NET was comprehensively evaluated on four scRNA-seq datasets with distinct drug treatments. The results indicate that the model excels in predicting drug response labels and identifying relevant gene features, particularly in binary datasets comprising sensitive and resistant cells. Furthermore, ATSDP-NET successfully identified key genes (KGs) associated with cisplatin response in oral squamous cell carcinoma, underscoring its practical value in elucidating the molecular mechanisms underlying drug resistance.

## Data Availability

The original contributions presented in the study are included in the article/Supplementary material, further inquiries can be directed to the corresponding authors.
