# Supplementary figures and images for: A drug response prediction method for single-cell tumors combining attention networks and transfer learning
Source: Front Med (Lausanne). 2025 Aug 21;12:1631898. doi: 10.3389/fmed.2025.1631898 (PMC12408664; doi:10.3389/fmed.2025.1631898)

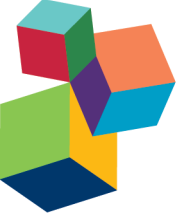

frontiers

Supplement: Supplementary file 1 [file Supplementary_file_1.zip › logo1.pdf]

A

frontiers  
FOR YOUNG MINDS

B

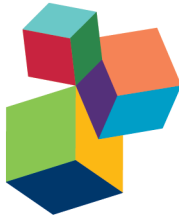

Supplement: Supplementary file 1 [file Supplementary_file_1.zip › logos.pdf]

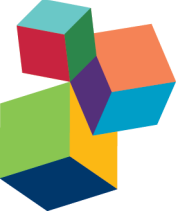

Supplement: Supplementary file 1 [file Supplementary_file_1.zip › logo2.pdf]

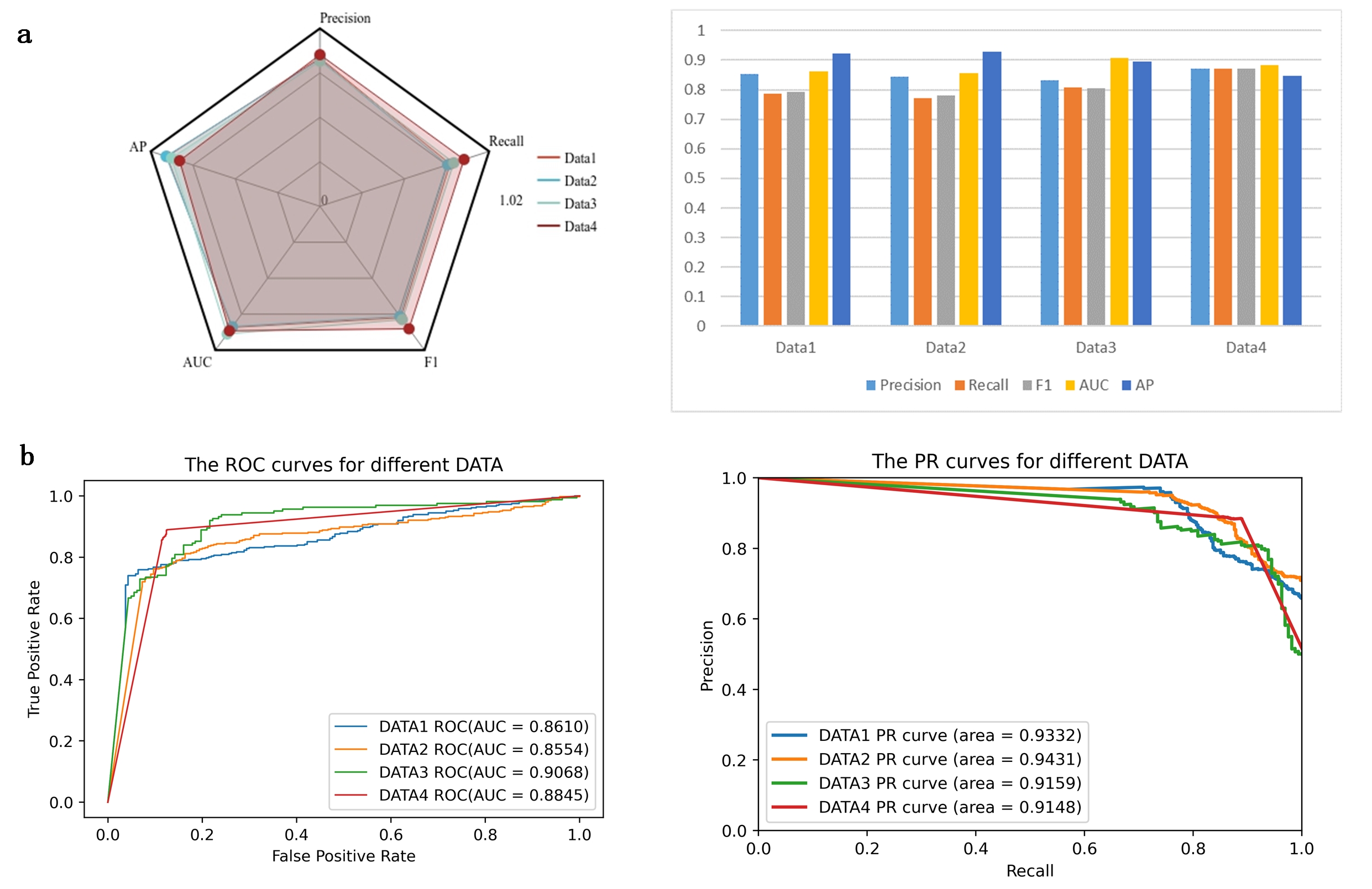

Supplement: Supplementary file 1 [file Supplementary_file_1.zip › PDF examples/figure2.jpg]

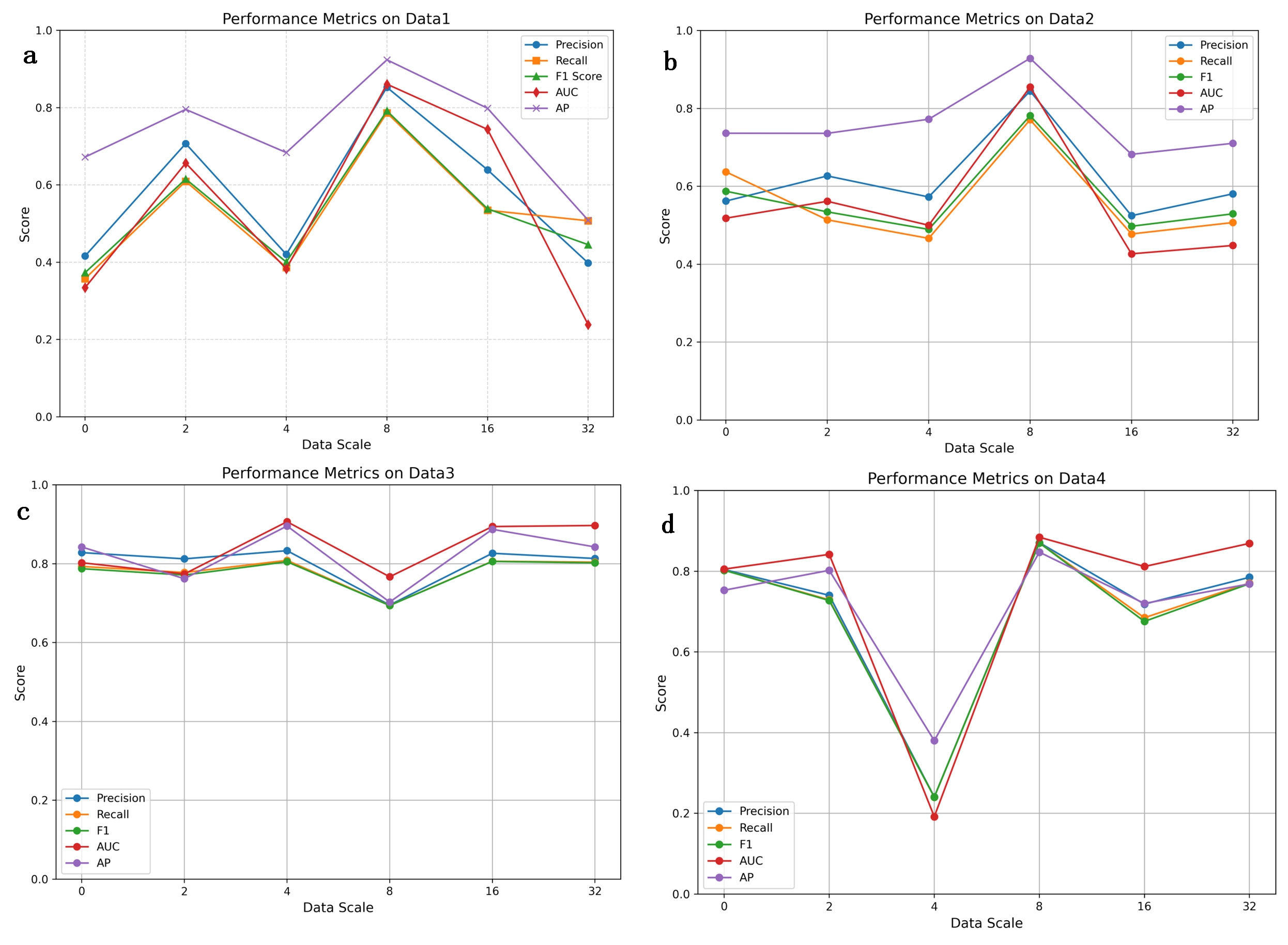

Supplement: Supplementary file 1 [file Supplementary_file_1.zip › PDF examples/figure3-old.jpg]

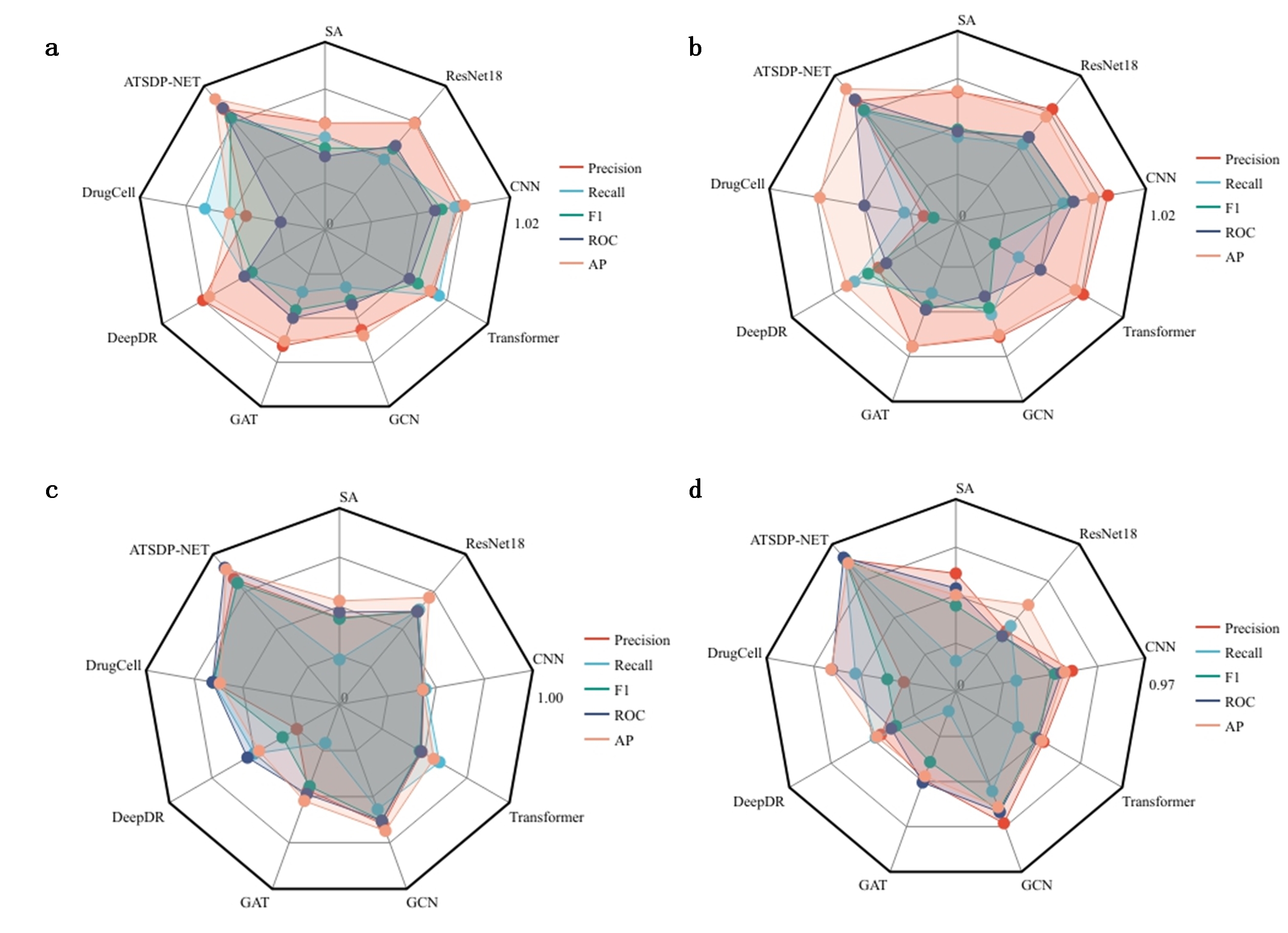

Supplement: Supplementary file 1 [file Supplementary_file_1.zip › PDF examples/figure4.jpg]

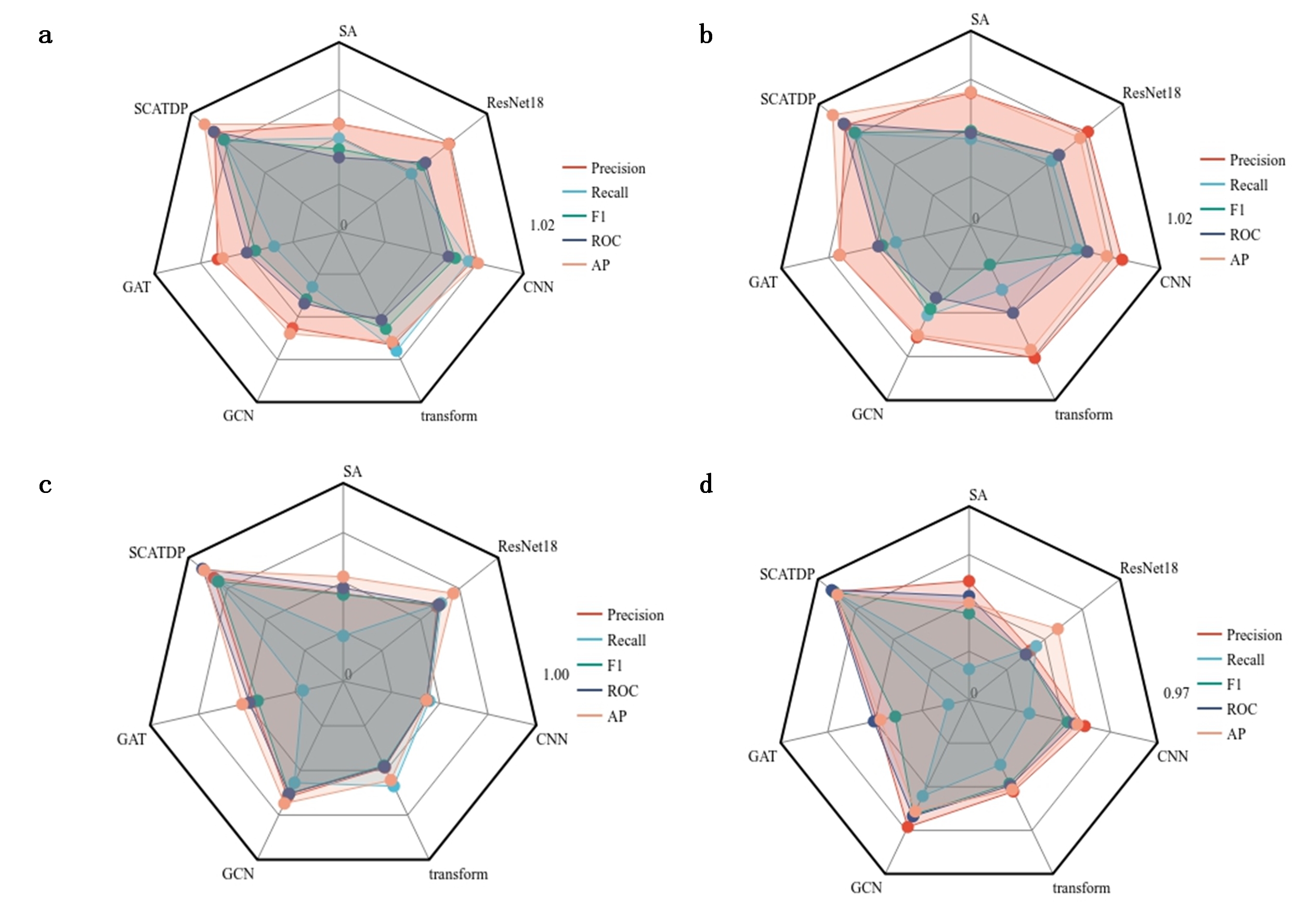

Supplement: Supplementary file 1 [file Supplementary_file_1.zip › PDF examples/figure4-old.jpg]

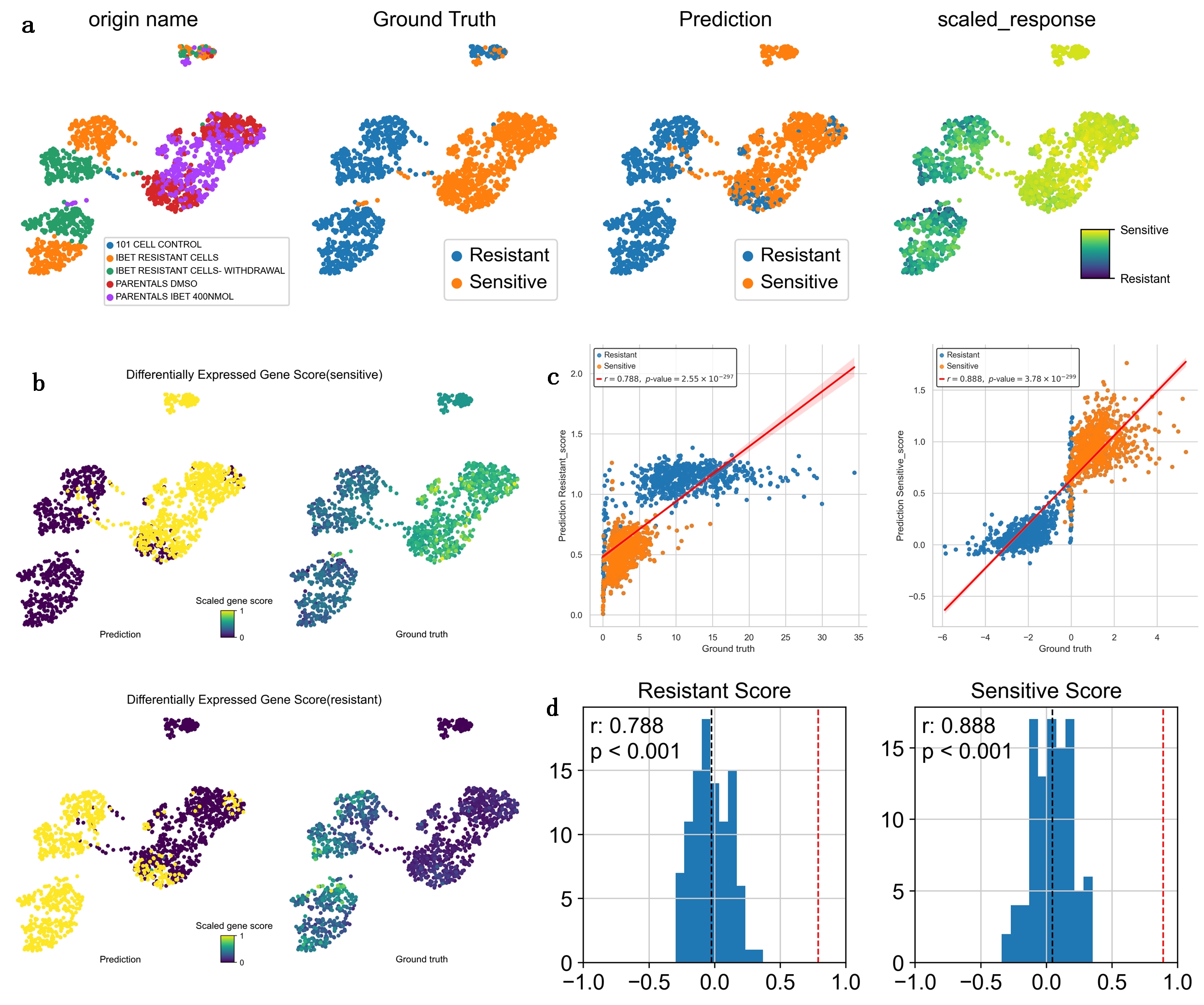

Supplement: Supplementary file 1 [file Supplementary_file_1.zip › PDF examples/figure5.jpg]

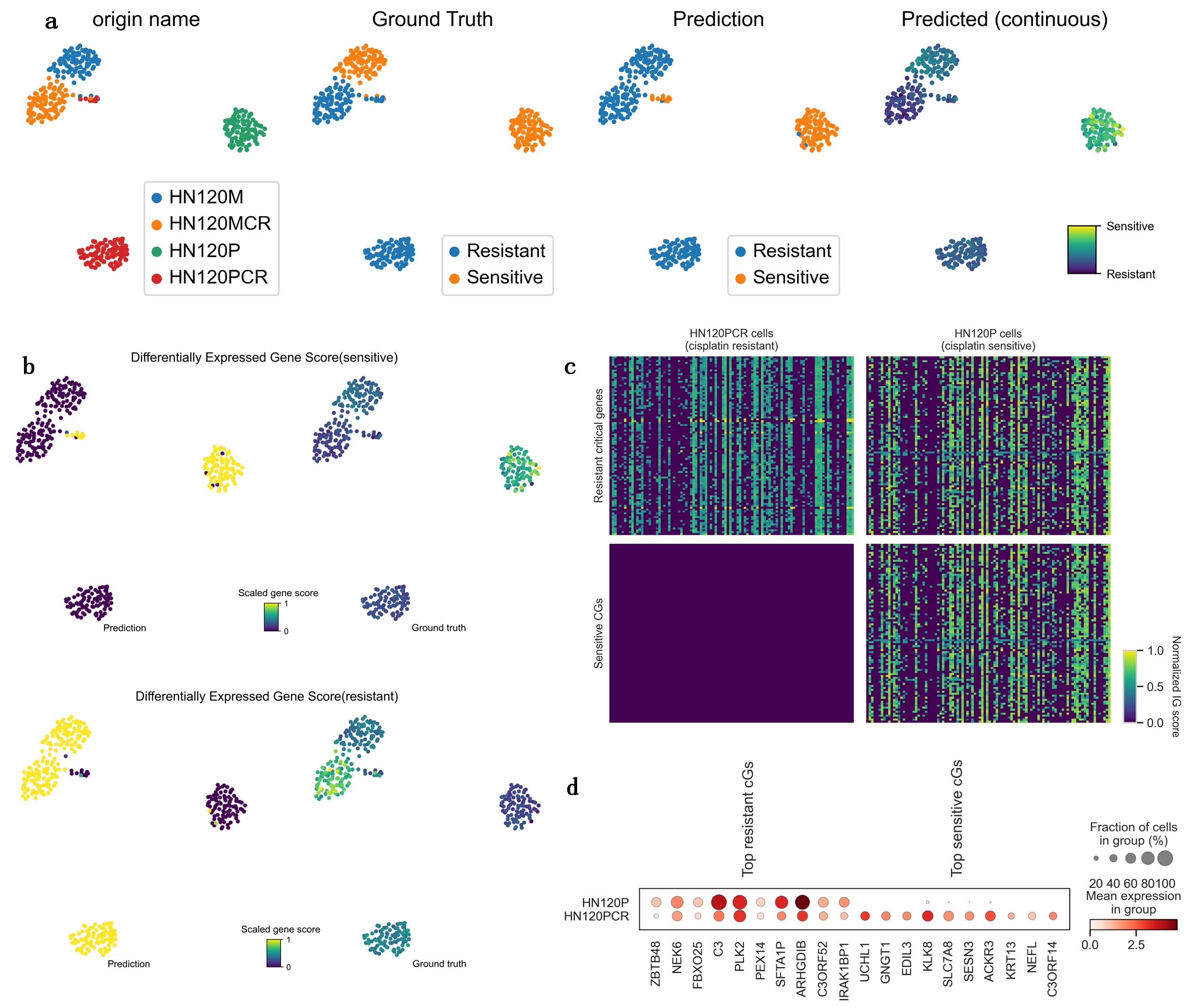

Supplement: Supplementary file 1 [file Supplementary_file_1.zip › PDF examples/figure6.jpg]

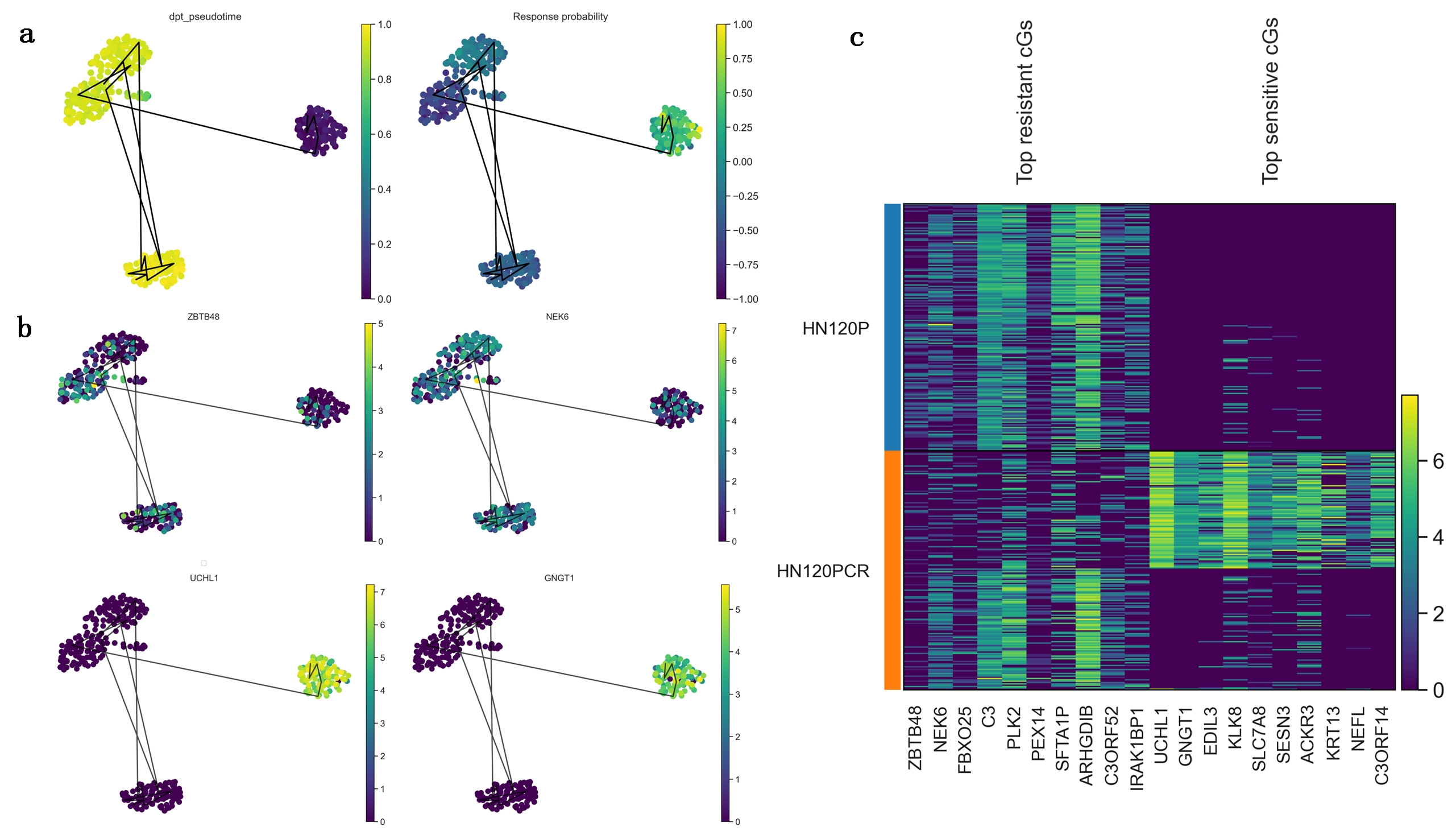

Supplement: Supplementary file 1 [file Supplementary_file_1.zip › PDF examples/figure7.jpg]

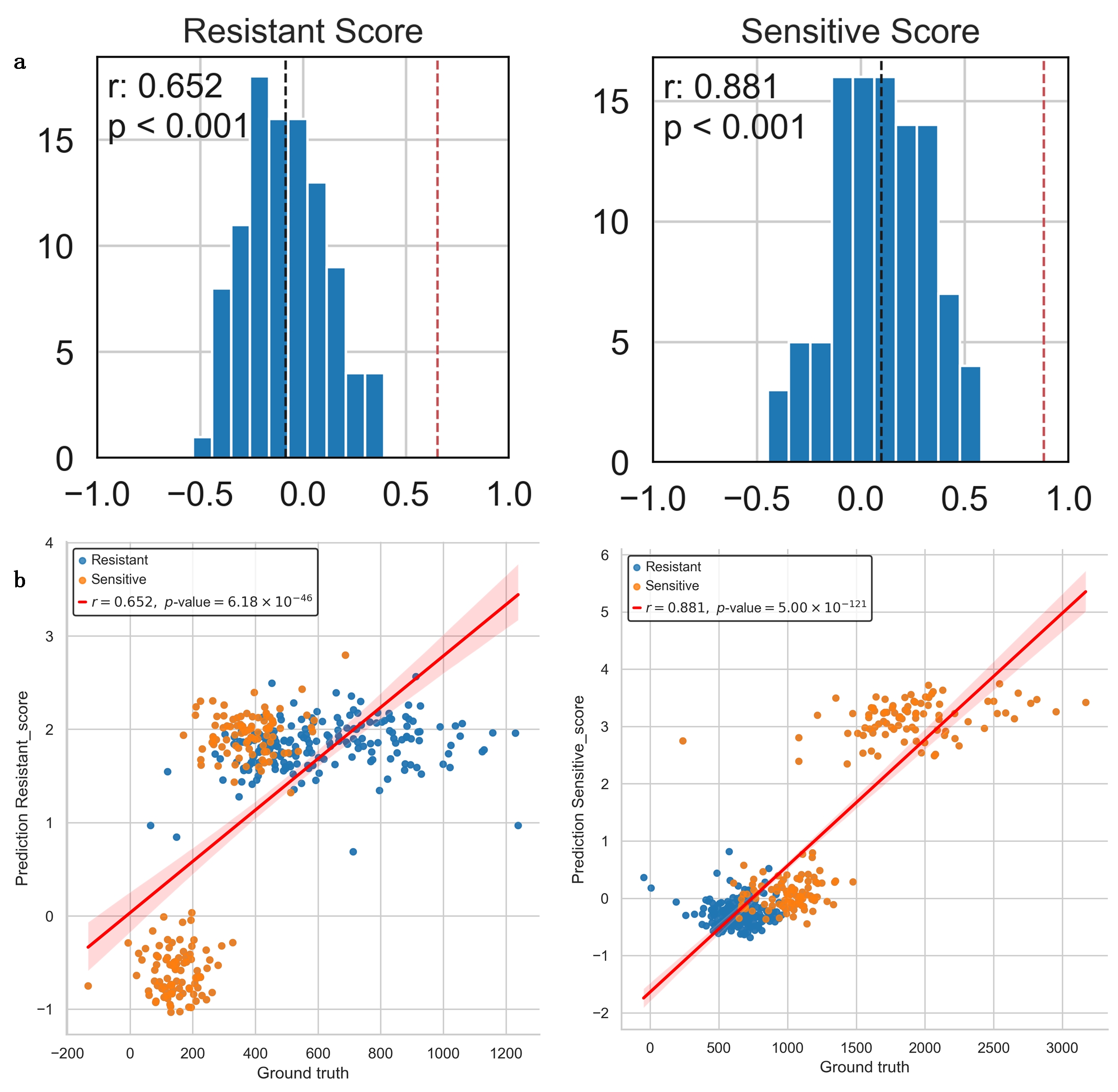

Supplement: Supplementary file 1 [file Supplementary_file_1.zip › PDF examples/figure8.jpg]

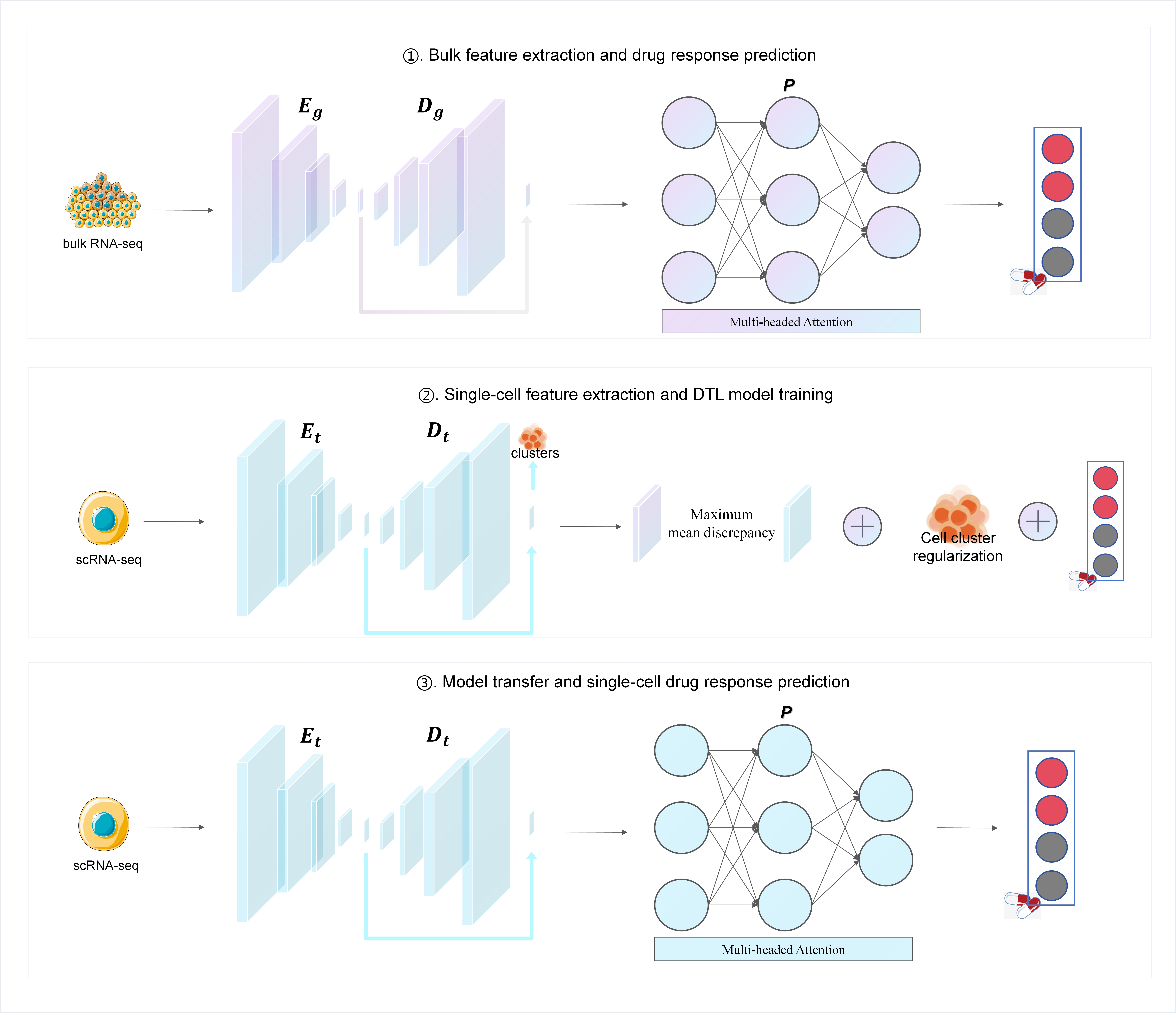

Supplement: Supplementary file 1 [file Supplementary_file_1.zip › PDF examples/Framework.jpg]

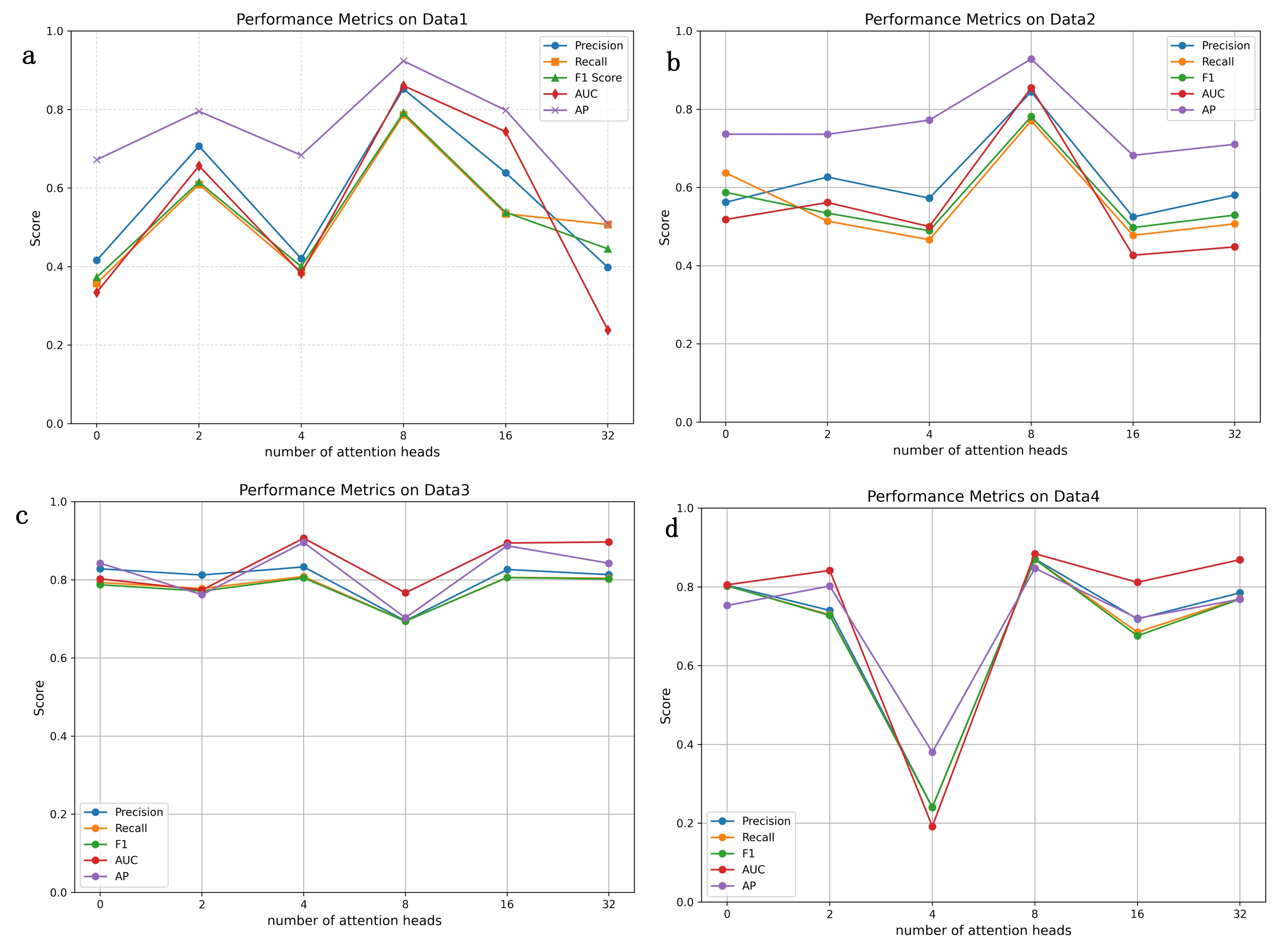

Supplement: Supplementary file 1 [file Supplementary_file_1.zip › PDF examples/figure3.jpg]
